# Chronic Opioid Treatment Arrests Neurodevelopment and Alters Synaptic Activity in Human Midbrain Organoids

**DOI:** 10.1002/advs.202400847

**Published:** 2024-03-28

**Authors:** Hye Sung Kim, Yang Xiao, Xuejing Chen, Siyu He, Jongwon Im, Moshe J. Willner, Michael O. Finlayson, Cong Xu, Huixiang Zhu, Se Joon Choi, Eugene V. Mosharov, Hae‐Won Kim, Bin Xu, Kam W. Leong

**Affiliations:** ^1^ Department of Biomedical Engineering Columbia University New York NY 10027 USA; ^2^ Institute of Tissue Regeneration Engineering (ITREN) Dankook University Cheonan 31116 Republic of Korea; ^3^ Mechanobiology Dental Medicine Research Center Dankook University Cheonan 31116 Republic of Korea; ^4^ Department of Physics Tsinghua University Beijing 100084 China; ^5^ Single Cell Analysis Core JP Sulzberger Columbia Genome Center Columbia University Irving Medical Center New York NY 10032 USA; ^6^ Department of Psychiatry Columbia University Medical Center New York NY 10032 USA; ^7^ Division of Molecular Therapeutics New York State Psychiatric Institute New York NY 10032 USA; ^8^ Department of Nanobiomedical Science and BK21 PLUS NBM Global Research Center for Regenerative Medicine Dankook University Cheonan 31116 Republic of Korea; ^9^ Department of Systems Biology Columbia University Irving Medical Center New York NY 10032 USA

**Keywords:** brain organoid, fentanyl, neurodevelopment, opioid, single‐cell RNA sequencing

## Abstract

Understanding the impact of long‐term opioid exposure on the embryonic brain is critical due to the surging number of pregnant mothers with opioid dependency. However, this has been limited by human brain inaccessibility and cross‐species differences in animal models. Here, a human midbrain model is established that uses hiPSC‐derived midbrain organoids to assess cell‐type‐specific responses to acute and chronic fentanyl treatment and fentanyl withdrawal. Single‐cell mRNA sequencing of 25,510 cells from organoids in different treatment groups reveals that chronic fentanyl treatment arrests neuronal subtype specification during early midbrain development and alters synaptic activity and neuron projection. In contrast, acute fentanyl treatment increases dopamine release but does not significantly alter gene expression related to cell lineage development. These results provide the first examination of the effects of opioid exposure on human midbrain development at the single‐cell level.

## Graphical abstract

A human midbrain model that uses human‐induced pluripotent stem cell‐derived midbrain organoids is established and applied to assess cell‐type‐specific responses to acute and chronic fentanyl treatment and withdrawal.

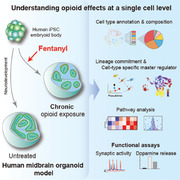

## 1 Introduction

The opioid epidemic has reached crisis levels globally, with opioid use disorder (OUD) affecting 40 million people worldwide.^[^ ^1^ ^]^ Misuse of prescription opioids, heroin, and potent fentanyl derivatives triggered a tenfold increase in overdose fatalities in the United States from 2013 to 2018.^[^ ^2^ ^]^ The COVID‐19 pandemic has worsened this situation, with significant increases in opioid use, misuse, and overdoses since early 2020.^[^ ^3^ ^]^ The increased opioid use unfortunately includes greater prescription opioid use among pregnant women.^[^ ^4^, ^5^ ^]^ In surveys, 22–30% filled a prescription for an opioid analgesic during pregnancy, and 21.2% reported misuse.^[^ ^5^, ^6^ ^]^ Opioid use during pregnancy leads to adverse maternal outcomes and adverse effects of prenatal exposure to opioids in utero.^[^ ^7^ ^]^

Prenatal opioid exposure includes the use and misuse of prescription and illicit opioid drugs, causing deleterious symptoms in neonates, including neonatal abstinence syndrome (NAS), small head circumference,^[^ ^8^ ^]^ decreased cerebral volume,^[^ ^9^ ^]^ microstructural brain injury,^[^ ^5^ ^]^ and low birth weight.^[^ ^4^, ^5^, ^10^ ^]^ Extensive clinical data suggest that in utero opioid exposure adversely affects developing organ systems, including the central nervous system.^[^ ^5^, ^11^ ^]^ Impaired neurodevelopment of neonates is correlated with long‐term problems with cognitive, behavioral, and developmental outcomes in childhood, as indicated by follow‐up studies of individuals who had in utero opioid exposure.^[^ ^12^ ^]^ However, the cellular and molecular mechanisms by which opioids disrupt neurodevelopment remain unclear. A better understanding of the risks of long‐term opioid exposure is especially urgent because synthetic opioids such as fentanyl, methadone, and buprenorphine are frequently used to treat pregnant patients with OUD and neonates with NAS.^[^ ^13^ ^]^

Studying the effects of opioids on human neurodevelopment is challenging due to limited accessibility to fetal brain specimens, ethical conflicts, and complex individual drug abuse histories. Preclinical studies using animal models have revealed detrimental effects of opioid use on the neurodevelopment of offspring,^[^ ^14^ ^]^ but differences between animal species and humans have led to a gap in neuropsychiatric clinical translation and failures in drug development.^[^ ^15^ ^]^ Pharmacological therapeutics developed using animal models have been largely ineffective for treating opioid abuse in humans because 1) different species vary in their neurodevelopmental trajectories, receptor expression, and central opioid pharmacokinetics; and 2) animal models cannot capture the broad network of symptoms and environmental/social factors that are fundamental to human drug abuse and addiction.^[^ ^16^, ^17^ ^]^ Human postmortem brain specimens are used to assess human‐specific differences in gene expression, biomarkers, and neuroanatomy in drug abuse,^[^ ^18^, ^19^ ^]^ but cellular and molecular responses cannot be evaluated in a time‐dependent manner.

Brain organoids are excellent candidates for bridging the gap between animal models and human studies. Brain organoids are self‐assembled 3D cell aggregates that resemble the human fetal brain, generated in vitro from human pluripotent stem cells.^[^ ^20^ ^]^ Unlike conventional 2D cell cultures, 3D organoid models better recapitulate the fetal brain in cell composition, architecture, and lineage trajectory.^[^ ^21^, ^22^ ^]^ Brain organoids provide an unparalleled platform for the manipulation of neural tissue, enabling systematic studies of human neurodevelopment, disease modeling, and drug screening.^[^ ^22^, ^23^, ^24^, ^25^, ^26^ ^]^ Recent advances in guided organoid generation methods using lineage‐specific patterning factors allow the generation of microtissue that mimics specific brain regions, such as the cerebral cortex,^[^ ^27^, ^28^ ^]^ hippocampus,^[^ ^29^ ^]^ and midbrain.^[^ ^23^ ^]^ Characterization of brain development is being revolutionized by the use of brain organoids in combination with patient‐derived iPSC engineering, genetic engineering, epigenetics, and single‐cell multiomics.^[^ ^30^, ^31^ ^]^ Single‐cell RNA sequencing (scRNA‐seq) provides insights into underlying molecular signatures associated with early events in neurogenesis and synaptogenesis that are otherwise inaccessible to experimentation.^[^ ^25^, ^32^, ^33^ ^]^ However, cellular changes in human neuronal cell subtypes upon opioid exposure have not been reported. Several brain organoid models have been validated, but no brain organoid model has been established to explore the temporal and spatial changes of neurons in response to a stressor such as opioids.

A major source of dopamine in the brain is localized in the ventral midbrain, specifically the ventral tegmental area (VTA) and substantia nigra. The mesolimbic dopamine projection from the VTA to the nucleus accumbens in the forebrain is a key pathway for reward‐driven learning. In the adult brain, chronic opioid exposure alters dopamine signaling, promoting addiction and vulnerability to relapse. However, little is known about the influence of chronic opioid exposure on midbrain development. Here, we establish a human midbrain organoid model and apply it to investigate the effects of chronic opioid exposure on neurodevelopment at a molecular and cellular level. Using an organoid generation protocol described previously,^[^ ^23^ ^]^ we validate the midbrain organoid model for studying neurodevelopment and evaluate cell type‐specific transcriptomic responses to fentanyl exposure in the organoids. We use molecular characterization to obtain pseudotime metrics of midbrain organoid development and perform an in‐depth analysis of affected gene expression and pathways related to neurodevelopment due to acute and chronic fentanyl exposure and fentanyl withdrawal. Our investigation of neurodevelopment in fentanyl‐treated organoids will contribute to the development of therapeutics for opioid abuse and prenatal opioid exposure.

## 2 Results

### 2.1 Generation and Characterization of Midbrain Organoids

**Figure 1:**
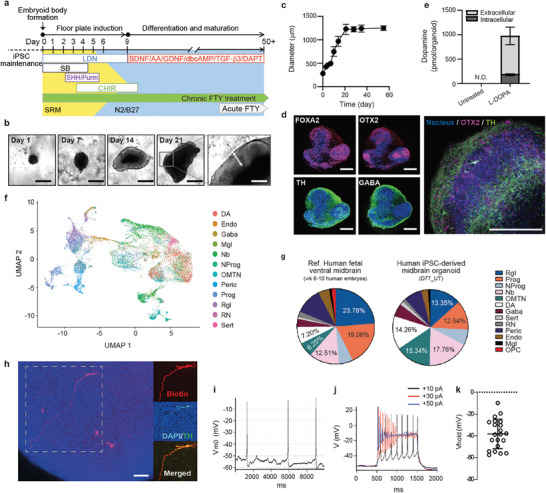
Generation and characterization of midbrain‐like organoids. a) Timeline of midbrain‐like organoid development. b) Morphological changes and c) growth (*n* = 20 organoids, three independent experiments) of organoids over time (scale bars, 500 µm for day 1–21, 200 µm for inset). d) Cytoarchitecture of organoids at day 21 (scale bar, 200 µm). e) Dopamine content in day 35 organoids before and after 1 h treatment with 100 µm L‐DOPA (*n* = 3 organoids, three independent experiments). f) Cell type identification of all samples (day 53 and day 77 organoids, *n* = 5 organoids/each condition, 25,510 cells sequenced). g) Cell type composition in human fetal midbrain (Reference data [36]) versus iPSC‐derived midbrain (this study). h–k) Electrophysiological properties of neurons in day 35 organoids. h) Recorded cells were labeled with biocytin dye (red) and added to the patch pipette. After fixation, organoids were immunostained for TH (green). All recorded neurons (*n* = 21) were confirmed to be TH^+^ (scale bar, 100 µm). i) Representative whole‐cell recording from spontaneously active DA neurons. j) Evoked action potentials following step current injections of +10 pA (black), +30 pA (red), and +50 pA (blue). k) Average resting membrane potential of DA neurons in organoids. See also Figure S1 (Supporting Information).

We generated midbrain organoids using a protocol adapted from Kriks et al. and Jo et al.^[^ ^23^, ^24^ ^]^ with minor modifications (**Figure** **1**a) and evaluated the early development, maturation, and functional assays at multiple time points (Figure S1a, Supporting Information). Human iPSCs were dissociated into single cells to form uniformly sized embryoid bodies (EBs) (≈300 µm in diameter) in untreated U‐bottom 96‐well plates (Figure 1b). During days 7–14, EBs showed uniform neuroectoderm formation along the outer surface, where the EBs were optically translucent and radially organized. After 9 days of neural induction, these neuroectoderm‐containing spheroids were cultured in N2 neuronal media supplemented with neurotrophic factors (brain‐derived neurotrophic factor, ascorbic acid, glial cell‐derived neurotrophic factor, cAMP, TGF‐β3, and the γ‐secretase inhibitor DAPT). After 21 days, the organoids grew to 0.8–1.2 mm in diameter (Figure 1c), and their cytoarchitectures were examined by immunocytochemical analysis (Figure 1d). Midbrain progenitor cells expressing orthodenticle homeobox 2 (OTX2) and forkhead box A2 (FOXA2) proteins were located closer to the organoid core, with OTX2 expression found in the apical surface and extending to the intermediate region of the neuroepithelia (Figure 1d; Figure S1b,c, Supporting Information). In contrast, dopaminergic (TH‐positive) and GABAergic (GABA‐positive) neurons were found along the outer edge of the organoids, similar to the layering in human midbrain development (Figure S1b,c, Supporting Information).^[^ ^34^ ^]^

To determine whether organoids increasingly resembled the human midbrain in terms of gene expression as they developed, we monitored the expression of marker genes for 35 days by qPCR (Figure S1d, Supporting Information). Expression of pluripotency markers (octamer‐binding transcription factor 4 (*OCT4*) and homeobox protein *NANOG*) decreased upon neuronal induction, while expression of pan‐neuronal markers (neuron‐specific class III beta‐tubulin (*TUJ1*) and microtubule‐associated protein 2 (*MAP2*)) increased. Expression of *OTX2*, a homeodomain transcription factor required for midbrain patterning,^[^ ^35^ ^]^ increased after 7 days of floor‐plate induction. We also observed co‐expression of the floor‐plate marker *FOXA2* and the roof‐plate marker *LMX1A*, a unique feature of midbrain development.^[^ ^24^ ^]^ We observed up‐regulation of various midbrain‐specific neuronal markers, including tyrosine hydroxylase (*TH*), dopamine transporter (*DAT*), and paired‐like homeodomain 3 (*PITX3*) during differentiation. In contrast, expression levels of forebrain (*PAX6*) and hindbrain (*TBR2* and *GBX2*) markers were low and unchanged. Although the expression level of *PAX6* increased over time, the fold change was not significant from that of genes related to floor plate markers (i.e., *FOXA2* and *LMX1A*) and midbrain‐specific markers (i.e., *TH*, *OTX2*, *DAT*, and *PITX3*). Immunocytochemical staining showed a decrease in OTX2^+^ cells and an increase in TH^+^ cells between days 7 and 35 of differentiation (Figure S1b, Supporting Information). Together, the cells of the midbrain organoids exhibited a gradual transition from proliferating neuroprogenitor cells into more mature midbrain‐specific neurons.

To determine the cell composition of the iPSC‐derived midbrain organoids, scRNA‐seq was performed, and data were analyzed in terms of cell‐type signatures of the human fetal ventral midbrain in 6–11‐week embryos as described by Manno et al.^[^ ^36^ ^]^ Cells from days 53 and 77 were analyzed to evaluate the maturation. At both time points, cells from 3 to 5 organoids in each treatment group were pooled. A total of 25 510 single cells were analyzed (Table S1, Supporting Information). We identified 12 cell types in the midbrain organoids, including progenitor cell types (Prog), radial glia‐like cells (Rgl), neural progenitors (NProg), neuroblasts (Nb), neuronal cell types (oculomotor and trochlear nucleus (OMTN), dopaminergic (DA), GABAergic (Gaba), red nucleus (RN), and serotoninergic (Sert) neurons), and mesoderm‐derived cell types (endothelial cells (Endo), pericytes (Peric), microglia‐like cells (Mgl)) (Figure 1f,g; Figure S1e–h, Supporting Information). The cell composition of the organoids was similar to that of the human fetal ventral midbrain at weeks 8–10, except that it lacked oligodendrocyte progenitor cells (OPC) as of day 77 of culture. From day 53 to day 77, the percentage of neuroblast and progenitor types decreased from 59% to 50%, and the percentage of neuronal and mesoderm‐derived cell types increased from 35% to 38% and from 6% to 12%, respectively (Figure S1e, Supporting Information). Together, these results indicate the differentiation of neuroprogenitor cells into neurons in the developing organoids.

To determine the cells’ electrophysiological properties, we immobilized the organoids on day 35 on a Matrigel‐coated coverslip and performed whole‐cell current‐clamp recordings of cells on or near the organoid surface. Biocytin dye was added to confirm that the cells were TH^+^ (Figure 1h). Electrophysiological analysis showed that 4 out of 21 recorded neurons fired spontaneous action potentials, with an average frequency of 0.6 ± 0.3 Hz (Figure 1i), which is a lower frequency than ≈4 Hz autonomous firing of mature mouse dopaminergic neurons. Injection of a +10 pA step current produced a train of stable action potentials, but higher current steps (+30 pA (red) and +50 pA (blue)) yielded action potentials with a higher frequency and lower amplitude that quickly ceased (Figure 1j). These results indicate that the dopaminergic neurons were not fully matured on day 35. The average resting membrane potential of the organoid neurons was ≈ −40 mV, which is higher than that of mature neurons (≈ −60 mV) (Figure 1k). Dopamine synthesis and release were undetectable in untreated organoids, but increased rapidly in the presence of the dopamine precursor L‐dihydroxyphenylalanine (L‐DOPA), demonstrating the ability of the neurons to produce neurotransmitters (Figure 1e). Notably, unlike immature organoids at day 35, organoids cultured for more than 90–180 days exhibited synchronized calcium signaling, indicating maturation in synaptic activity. Consequently, we could further investigate the inhibitory effect of fentanyl on neurotransmission within the synaptic networks of these organoids. Together, these results indicate that the midbrain organoids are similar to early‐stage human fetal midbrains, making them a potentially useful tool for understanding the effects of chronic opioid exposure on neurodevelopment.

### 2.2 Cellular Responses to Acute Opioid Exposure

**Figure 2:**
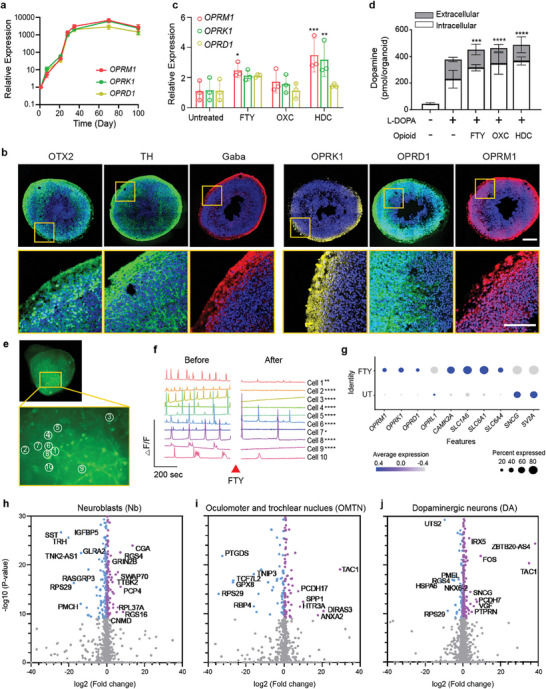
Cellular responses to acute opioid exposure. a) Gene expressions of opioid receptors, including mu (*OPRM1*), kappa (*ORPK1*), and delta (*OPRD1*), for 90 days of culture (*n* = 5–7 organoids, three independent experiments). b) ICC staining of dopaminergic neuronal markers and opioid receptors of day 35 organoids (scale bar, 200 µm (upper) and 100 µm (bottom)). c–j) Results of 4‐h treatment of opioids (FTY; fentanyl, OXC; oxycodone, HDC; hydrocodone) in midbrain‐like organoids at day 90. c) Alterations in gene expression levels of opioid receptors (*n* = 3 organoids, two independent experiments) and d) dopamine synthesis and release in response to 4‐h opioid treatment (*n* = 3 organoids, three independent experiments). Statistical analysis was determined by one‐way ANOVA as compared to untreated control (^*^ *p *< 0.05, ^**^ *p *< 0.01, ^***^ *p *< 0.001, and ^****^ *p *< 0.0001). e,f) Calcium imaging using Fluo4‐AM of Day 180 organoids showing decreased frequency of calcium transients after fentanyl treatment (Movie S1, Supporting Information). Statistical analysis of frequency and amplitudes was determined by *t*‐test (^*^ *p *< 0.05, ^**^ *p *< 0.01, and ^****^ *p *< 0.0001). Frequencies of all cells were significantly decreased after the FTY treatment (^***^ *p *< 0.001). g) Summarized changes in the expression of opioid receptors (*OPRK1*, *OPRL1*, *OPRM1*, and *OGFR*), second messenger effects of Ca^2+^ (*CAMK2A*), neurotransmitter transporters (*SLC1A6*, *SLC6A1*, and *SLC6A4*), and synaptic plasticity (*SNCG* and *SV2A*). Each dot color and size represent the relative gene expression level and proportion of expressing cells, correspondingly. h–j) Volcano plots of gene expression changes in three cell types after fentanyl treatment. Blue: UT, purple: FTY. See also Figure S2 (Supporting Information).

We confirmed that the organoids express opioid receptors, including mu (OPRM1), kappa (OPRK1), and delta (OPRD1). mRNA expression levels of opioid receptors increased until day 35, then saturated (**Figure** **2**a). Opioid receptors were detected in the same locations as TH^+^ or GABA^+^ neurons (Figure 2b).

Acute opioid administration is used for pain relief during labor and delivery. To investigate the impact of short‐term opioid treatment on the fetal brain (similar to acute opioid exposure in utero), midbrain organoids were treated with synthetic opioids, fentanyl, oxycodone, and hydrocodone for 4 h (Figure S2a,b, Supporting Information). Clinically relevant doses of opioids ranging from 4 to 74 nm were tested.^[^ ^37^ ^]^ This treatment resulted in a two to fourfold increase in the mRNA expression of the three opioid receptors relative to untreated organoids (Figure 2c). Furthermore, intracellular and extracellular dopamine levels also increased in organoids treated for 4 h with opioids followed by 1 h treatment with L‐DOPA (Figure 2d). Spontaneous calcium transients were attenuated in both frequency and amplitude in cells of fentanyl‐treated organoids, based on measurements using Fluo‐4 AM (Figure 2e,f; Movie S1, Supporting Information). This finding indicates an attenuation of synchronized neurotransmission in organoids due to fentanyl exposure, potentially offering insights into the opioid modulation of pain perception.^[^ ^38^ ^]^

We performed single‐cell transcriptome analysis of fentanyl‐treated organoids (scRNA sample 'D53_UT' versus 'D53_AC_FTY'). We observed that the 4‐h fentanyl treatment altered the gene expression profiles of neuroblasts (Nb), oculomotor and trochlear nucleus (OMTN) cells, and dopaminergic neurons (DA) (Figure 2h–j; Figure S2c,d, Supporting Information), but not of other neuronal cell types such as Gaba, Sert, and red nucleus (RN) cells. Interestingly, upon analyzing the single‐cell expression of opioid response genes,^[^ ^18^, ^39^ ^]^ we observed a noteworthy decrease in the expression of synaptic plasticity genes (*SNCG* and *SV2A*) in the fentanyl‐treated group (Figure 2g). This reduction in expression levels suggests that acute opioid treatment may lead to a decrease in synapse transmission. Gene set enrichment analysis, however, showed no significant change in biological pathways (Figure S2d, Supporting Information). We reasoned that a 4‐h acute opioid exposure did not post observable transcriptomic profile changes. Together, these results suggest that acute opioid exposure elicits cell‐type‐specific responses in the midbrain but does not significantly alter gene profiles in neuronal lineage specification and neuron projection.

### 2.3 Chronic Fentanyl Treatment Impairs Neurodevelopment

**Figure 3:**
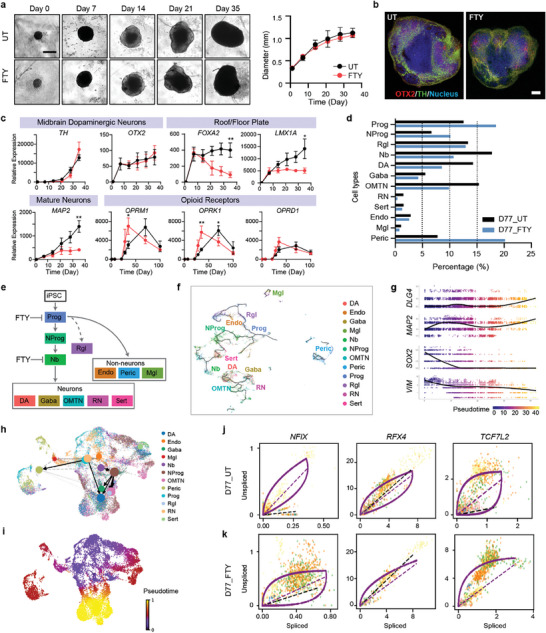
Chronic fentanyl treatment alters cell heterogeneity in developing midbrain organoids. a) Timeline of specimens showing organoid size and morphology (scale bar, 500 µm). Profiles of organoid growth (*n* = 20 organoids, three independent experiments). b) Spatial characterization of the midbrain patterning marker (OTX2) and function protein (TH) in day 21 midbrain organoids (scale bar, 250 µm). c) Fold changes of target genes of roof/floor plate (*FOXA2* and *LMX1A*), dopaminergic neurons (*TH* and *OTX2*), mature neurons (*MAP2*), and opioid receptors (*OPRM1*, *OPRK1*, and *OPRD1*) (*n* = 5–7 organoids per group/batch, three independent experiments) (*t*‐test; ^*^ *p *< 0.05, and ^**^ *p *< 0.01). d) Bar chart depicting the percentage of cells in untreated or fentanyl‐treated midbrain organoids at day 77. e) Hypothesized hierarchy of neural cell development. Fentanyl affected cell fate transition in progenitors and neuroblasts. f) Single‐cell lineage trajectory as plotted by similarity‐based analysis (Monocle3 algorithm). g) Trend of mature neuron markers (*DLG4* and *MAP2*) and stem cell markers (*SOX2* and *VIM*) of single cells in pseudotime (Monocle3 algorithm). h) Predicted cell fate transition by RNA velocity (scVelo algorithm). i) Predicted pseudotime state of single cells by RNA velocity. j,k) Phase portrait showing the levels of unspliced and spliced mRNA in untreated (D77_UT) or fentanyl‐treated organoids (D77_FTY). Above or below the purple dashed line indicates increasing or decreasing expression of a gene. See also Figure S3 (Supporting Information).

Human neuroimaging and animal studies have shown adverse effects of prenatal opioid exposure on neurodevelopment,^[^ ^17^, ^40^ ^]^ including dysregulated functional connectivity, decreases in brain volume, and delayed neurodevelopment.^[^ ^12^, ^41^ ^]^ We sought to investigate the effects of chronic fentanyl treatment on neurodevelopment using our midbrain organoid model. Organoids were treated with fentanyl for 53 or 77 days, starting from the first day of floor‐plate induction. Given that the fentanyl dose ranging from 0.8 to 200 ng mL^−1^ (equivalent to 0.3–74 nm) in vitro corresponds to serum concentration levels observed in high‐dose fentanyl applications in both adults and neonates,^[^ ^42^ ^]^ we chose a concentration of 74 nm fentanyl as the clinically relevant dose for this study. We examined the morphology of the midbrain organoid over time and found that the tissue developed into rosette‐like regions, but chronic fentanyl exposure did not alter the gross morphology or organoid size over 77 days of culture (**Figure** **3**a), possibly because organoid growth reached an upper limit due to the lack of a supporting 3D hydrogel scaffold and a perfusable vasculature. We observed no significant changes in the distribution of OTX2 and TH proteins in fentanyl‐treated organoids as compared to untreated ones at the early development stage (Figure 3b). In mature organoids, MAP2 and TH were abundantly expressed in both organoids, and their structural organization was not significantly distinguished (Figure S3a, Supporting Information).

We next explored whether differentiation and maturation of various cell types were affected by chronic opioid exposure. Interestingly, the expression levels of the midbrain progenitor markers *FOXA2* and *LMX1A* and the pan‐neuronal marker *MAP2* were lower in the chronic fentanyl treatment group than in untreated organoids (Figure 3c). In contrast, there was no significant difference in the expression of genes related to pluripotency (*OCT4* and *NANOG*) or the midbrain (*TH*, *OTX2*, *DAT*, and *PITX3*), forebrain (*PAX6*), or hindbrain (*TBR2* and *GBX2*) at the bulk mRNA level as measured by qPCR (Figure S3b, Supporting Information). All three opioid receptors were expressed earlier in the chronic fentanyl group (with a peak at day 35) than in the untreated group (peak at day 70) (Figure 3c).

We examined scRNA‐seq data to characterize how fentanyl affected midbrain neuronal differentiation. A lower percentage of mature neurons (DA, Gaba, OMTN, RN, and Sert) and a higher percentage of pericytes (Peric) and progenitor cells (Prog and NProg) were observed in the fentanyl‐treated samples (D77_FTY) than in the untreated samples (D77_UT) (Figure 3d; Figure S1e, Supporting Information). Mature dopaminergic neurons accounted for 14.3% of cells in the D77_UT sample and only 8.6% of cells in the D77_FTY sample. However, although fewer mature neurons developed in the fentanyl‐treated samples, the average level of TH remained similar. We hypothesized that this change in cell composition resulted from alterations in cell lineage decisions in midbrain development (Figure 3e). To understand the chronology of neurodevelopment, we constructed developmental cell fate trajectories using two state‐of‐the‐art lineage decision methods: Monocle 3 (a similarity‐based method; Figure 3f,g) and scVelo (an RNA velocity‐based method^[^ ^32^, ^43^ ^]^; Figure 3h–k). In Monocle 3, UMAP^[^ ^44^ ^]^ visualization of the overall dataset showed two distinct groups: progenitors (Prog, NProg, Rgl, and Nb) and neurons (DA, Gaba, OMTN, and RN) (Figure 3f; Figure S3e–g, Supporting Information). Pseudotime lineage analysis showed dynamic gene profile changes over pseudo‐age. For example, expression levels of stem cell genes (*SOX2* and *VIM*) and mature neuron markers (*MAP2* and *DLG4*) showed an inverse relationship (Figure 3g; Figure S3h–j, Supporting Information). We calculated RNA dynamics (rates of transcription, splicing, and degradation of individual genes) based on the mapped spliced and unspliced mRNA reads with scVelo (Figure S3k,l, Supporting Information). By quantifying the connectivity of cell clusters, partition‐based graph abstraction (PAGA) provided a simple abstract graph of cell fate connectivity (Figure 3h). We checked the pseudotime projection of the dataset and plotted velocity streamlines of RNAs in a normal brain organoid (Figure S3m, Supporting Information) and a growth‐impaired organoid (Figure S3n, Supporting Information). In theory, quiescent stem cells and terminally differentiated cells (e.g., neurons) show close to zero velocity values as they are at a steady state of spliced and unspliced mRNA levels. The size of the stream line arrows was proportional to the cell's velocity. We observed low‐velocity fields in Prog and Rgl cells, as well as in Gaba and DA cells (Figure S3m,n, Supporting Information), consistent with previous studies.^[^ ^32^, ^45^ ^]^ Phase portraits of neuronal specification markers and mature neuron function markers showed that chronic fentanyl exposure arrested cells at an early stage of neurodevelopment (Figure 3j,k; Figure S3o–q, Supporting Information). Although radial glia‐like cells were identified in the midbrain organoids, their specification and ability to give rise to neurons are not clear.

The trajectory maps illustrated that 1) neuron specification and maturation were affected by chronic fentanyl exposure, and more cells were arrested as Prog or Nb cells instead of becoming neurons (Figure 3d,e); 2) distinct neuron lineages converged back to intermediate progenitors (neuroblasts); 3) iPSCs gave rise to both mesoderm‐ and ectoderm‐derived cells in midbrain organoids. Together, these results suggest that the midbrain organoid model provides a useful framework for investigating cell lineage decisions and driver gene dynamics in neurodevelopment.

### 2.4 Chronic Fentanyl Exposure Alters Synaptic Plasticity in Neurons

**Figure 4:**
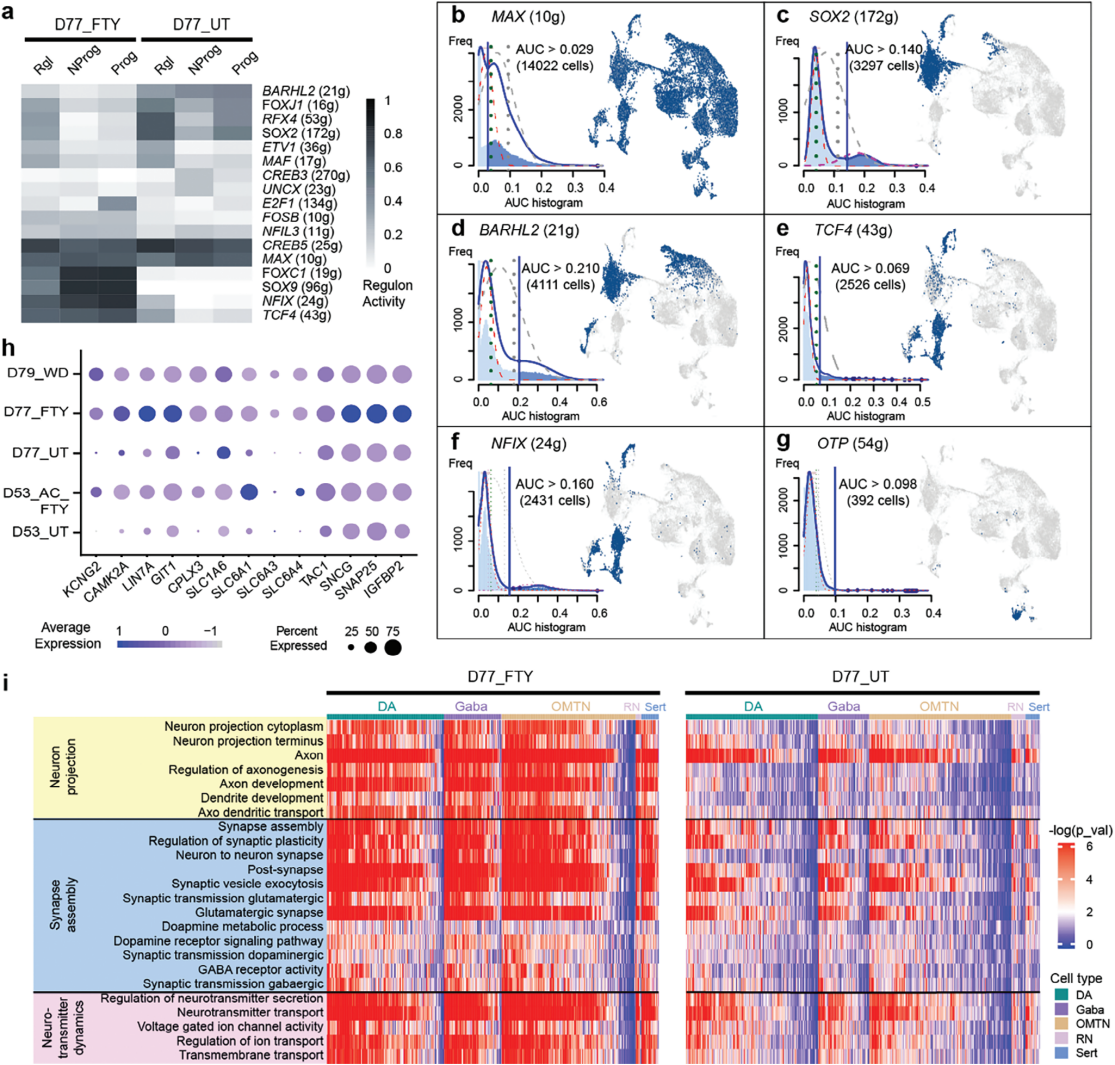
Chronic fentanyl exposure alters regulon networks and synaptic activity. a) Identified master regulators in each cell type based on regulon analysis (SCENIC algorithm). The number of predicted target genes was given for each transcription factor in the bracket. b–g) Activity score of each regulon network and its binarized activity mapped in UMAP. The blue vertical line in the AUC histogram yielded the cutoff for the “ON” or “OFF” state of that regulon network. The blue dots in UMAP indicated the cells with an "ON" state. See Figure S1g for the distribution of D77_UT and D77_FTY. Both samples (D77_UT and D77_FTY) showed high activity of the *MAX* regulon. The untreated sample (D77_UT) showed a higher percentage of cells with “ON” states of *SOX2*, *BARHL2*, and *OTP*, while the fentanyl‐treated sample (D77_FTY) had higher activities of early neurodevelopmental transcription, such as *TCF4* and *NFIX*. h) Dotplot showing individual gene expression levels and the percentage of expressing cells in the neurons of all five samples. A higher percentage of cells in D77_FTY and D53_AC_FTY were expressing genes related to ion transport (*KCNG2*), synaptic plasticity (*CAMK2A*, *LIN7A*, *GIT1*, and *CPLX3*), and neurotransmitter release (*SLC1A6*, *SLC6A1*, *SLC6A3*, and *SLC6A4*) as compared to untreated samples. i) Single‐sample gene set enrichment analysis (ssGSEA) showing altered pathways relating to neuron projection (yellow), synapse assembly and signal transduction (blue), and neurotransmitter dynamics (purple) in fentanyl‐treated samples (left heatmap) and untreated samples (right heatmap). See also Figure S4 (Supporting Information).

We analyzed the heterogeneous cell type‐specific response to chronic fentanyl exposure based on scRNA‐seq results. Differentially expressed genes between D77_UT and D77_FTY were identified by using the FindMarkers function in Seurat (**Figure** **4**a–g; Figure S4a–e, Supporting Information). Both samples expressed high levels of secretory‐ or vesicle‐associated proteins that regulate neurotransmitter release^[^ ^46^ ^]^ (*VGF*, *SCG2*, *VAMP2*, and *RAB3A*) in various neuronal types (DA, Gaba, OMTN, Sert, and RN) (Figure S4f–j, Supporting Information). A higher percentage of neurons (DA, Gaba, OMTN, Sert, and RN) in D77_FTY expressed genes related to neuronal functions such as ion transport (*KCNG2*), synaptic plasticity (*CAMK2A*, *LIN7A*, *GIT1*, and *CPLX3*), and neurotransmitter release (*SLC1A6*, *SLC6A1*, *SLC6A3*, and *SLC6A4*) (Figure 4h). Interestingly, opioid receptor genes (*OPRD1*, *OPRK1*, *OPKL1*, and *OPRM1*) did not show significantly different expression at a single‐cell level in the chronic fentanyl sample. Our ssGSEA analysis also identified pathways with gene set enrichment in each single cell (Figure 4i). In the D77_FTY sample, gene sets related to synaptic activity, neuron projection, and neurotransmitter transport were enriched in neurons (DA, Gaba, and OMTN). Differential responses between DA and Gaba were not observed (Figure 4i; Figure S4f, Supporting Information), likely because neural circuits were not yet well established in the organoids. Progenitor cells (Rgl, Prog, NProg, and Nb) in D77_UT exhibited high regulon activity in neuron specification and migration, while progenitor cells in D77_FTY showed high activity in neurogenesis, differentiation, and axonogenesis (Figure S4c, Supporting Information). These results indicate that progenitor cells in D77_FTY were arrested at an earlier stage than those in D77_UT.

Mesoderm‐derived cells (pericytes, endothelial cells, and microglia) in untreated midbrain organoids (D77_UT) were enriched with genes that mediate endothelium development, angiogenesis, and extracellular structure organization (Figure S4i, Supporting Information). In chronic fentanyl‐treated organoids, we observed elevated expression of genes related to immune response (*PLCG2*, *TFF3*, and *CRYAB*) and homeostasis (*EPYC* and *SPINK6*) (Figure S4j, Supporting Information). Interestingly, pericytes were responsive to fentanyl treatment, as demonstrated by significant transcriptome changes relative to untreated pericytes (Figures S2c and S4g, Supporting Information). The extracellular dopamine level of organoids with the chronic fentanyl treatment was higher than that of untreated organoids (Figure S3c, Supporting Information), but not significantly. Organoids with chronic fentanyl treatment also showed fewer neurons with calcium signaling (Figure S3d and Movie S2, Supporting Information), indicating that cell excitability and signal transmission were affected by the fentanyl treatment.

### 2.5 Neural Development Resumes in Transcriptomes When Fentanyl Is Withdrawn

**Figure 5:**
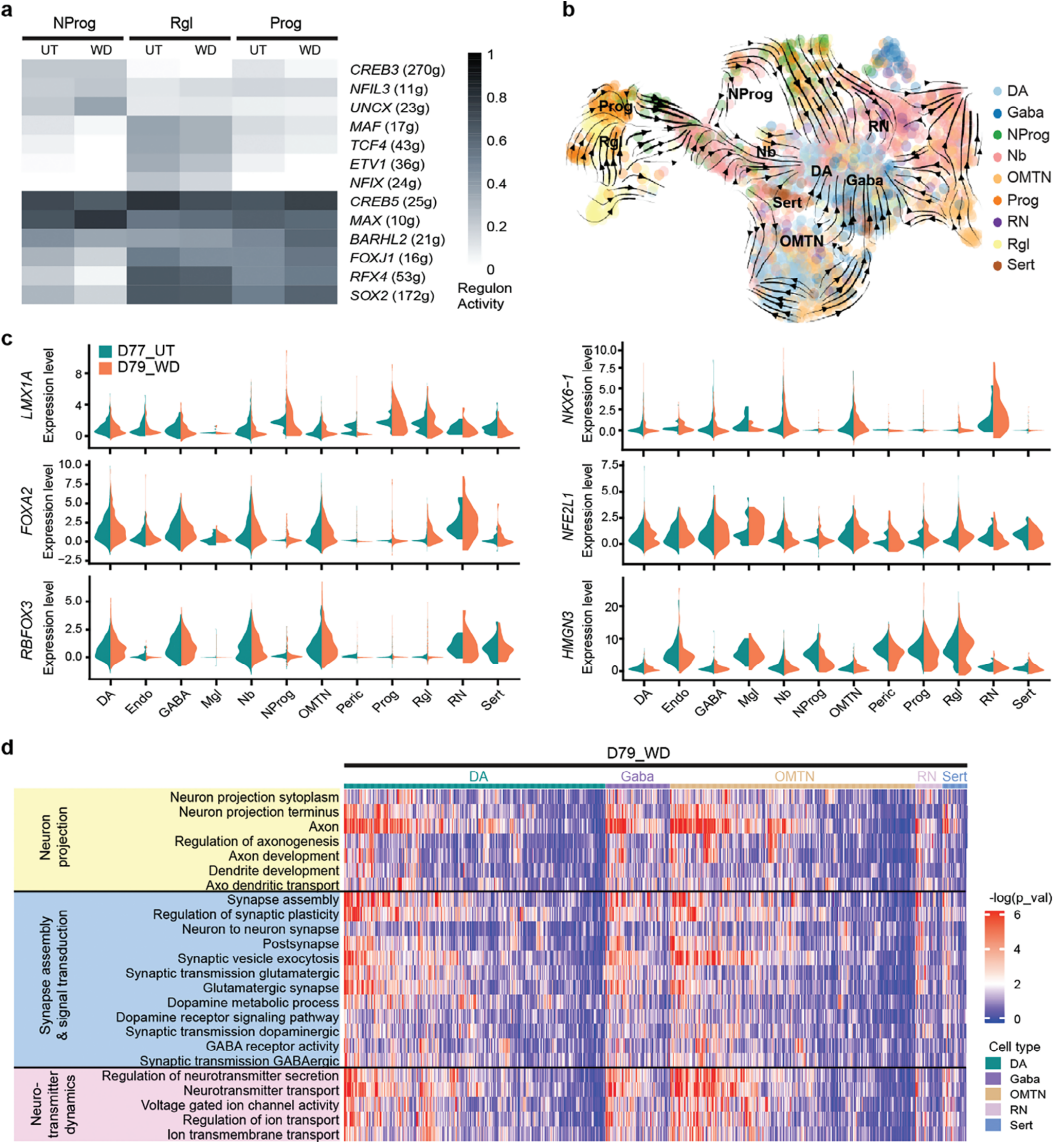
Neural development resumes in transcriptomes when fentanyl is withdrawn. a) Key master regulators identified in withdrawal sample (D79_WD) versus untreated sample (D77_UT) (SCENIC algorithm). b) RNA velocities were visualized as streamlines in a UMAP‐based embedding for all five samples. Spliced/unspliced mRNA dynamics disentangled cell lineage commitment at the single‐cell level. c) Violin plots show the gene expression level and percentage of expressing cells in D77_UT and D79_WD samples. d) Single sample gene set enrichment analysis (ssGSEA) shows pathways relating to neuron projection (yellow), synapse assembly and signal transduction (blue), and neurotransmitter dynamics (purple) restore basal states in fentanyl withdrawal samples. The pattern was more similar to untreated samples (D77_UT, right heatmap of Fig. 4I) than fentanyl‐treated samples (D77_FTY).

To determine whether the impacts of chronic fentanyl exposure on neurodevelopment can be reversed when fentanyl is withdrawn, we stopped fentanyl treatment for 2 days and then analyzed the cells. We performed differential expression analysis between the untreated (D77_UT) and withdrawal (D79_WD) samples and found that regulon activities related to neuronal differentiation and subtype specification (*FOXJ1*, *RFX4*, *SOX2*, and *BARHL2*) were substantially restored in progenitor cells (Prog, NProg, and Rgl) (**Figure** **5**a,b; Figure S5, Supporting Information). We also observed increased expression of the ventral midbrain progenitor gene *LMX1A*, subtype specification genes (*NKX6‐1* and *FOXA2*), and mature neuron markers (*RBFOX3*, *NFE2L1*, and *HMGN3*) in the D79_WD sample relative to D77_FTY (Figure 5c). Gene set enrichment analysis showed that the synaptic activity and neuron projection in D79_WD were more similar to D77_UT than to D77_FTY (Figure 5d), indicating that the synaptic activity decreased to a basal state in response to drug withdrawal. While we did not observe any functional recovery at the cellular level due to the lack of mature neurons, these results suggest that neural development resumes at the transcriptomic level when opioids are withdrawn.

## 3 Discussion

In utero opioid exposure impairs neurodevelopment and causes poor neurocognitive, behavioral, and developmental outcomes in childhood,^[^ ^12^ ^]^ but the molecular and cellular mechanisms underlying this dysregulation are unclear. One crucial neural circuit involved in opioid action in adults is the mesolimbic reward system. This system generates dopamine signals in the ventral tegmental area in the midbrain that propagate throughout the brain, playing a role in learning and reward pathways. Abused drugs, such as opioids, leverage this system to induce dopamine surges, providing relief from pain through acute drug exposure.^[^ ^47^ ^]^ Chronic opioid exposure disrupts dopamine signaling and induces transcriptional and epigenetic changes in brain regions within the mesolimbic system, thereby promoting addiction and vulnerability to relapse.^[^ ^47^, ^48^ ^]^ While opioid addiction is strongly associated with the mesolimbic system in the adult brain based on extensive clinical and preclinical data, this association in the developing human fetal brain remains poorly understood. We aimed to use organoids to investigate the molecular and cellular effects of chronic opioid exposure on the midbrain—the largest dopamine‐producing brain area and a region involved in the mesolimbic reward system during neurodevelopment.

Midbrain‐specific organoids were generated by a guided method using midbrain patterning molecules.^[^ ^23^, ^24^ ^]^ iPSC differentiation was directed towards floor‐plate lineages through the dual‐SMAD inhibition factors LDN 193 189 and SB431542, in combination with the Wnt pathway activator CHIR 99 021. Subsequently, floor‐plate cells were further directed towards ventral mesencephalic lineages through subsequent treatment with the small‐molecule agonist purmorphamine and recombinant SHH. The guided method used in this study has consistently shown reproducibility across various human iPSC lines and even ESCs, ensuring consistency and homogeneity at both morphological and transcriptional levels.^[^ ^49^, ^50^ ^]^ Consequently, iPSC‐derived midbrain organoids generated using this method have become a widely recognized choice for disease modeling and drug screening/toxicity testing applications.^[^ ^51^ ^]^ To validate the consistency of our approach, we compared the gene expression profiles of organoids across multiple independent batches generated using two iPSC lines (FA‐11 and WTC), and no significant variations were found (Figure S1i,j, Supporting Information), suggesting that our guided method reliably and consistently generates midbrain organoids.

The midbrain organoids exhibited cytoarchitecture and cell composition similar to those of the developing human ventral midbrain region.^[^ ^36^, ^52^ ^]^ The electrophysiological properties of the organoids at day 35 indicated that neurons at the organoid surface were not yet fully mature, as evidenced by their depolarized resting membrane potential, slow or absent pacemaking activity, and inability to maintain long action potential trains.^[^ ^27^, ^49^, ^53^ ^]^ The organoids model early stages of human embryonic development in which fully realized electrophysiological properties such as action potentials and spontaneous synaptic transmission are not yet observed and ontogenetic changes still occur.^[^ ^54^ ^]^ The electrophysiological properties of neurons in midbrain organoids gradually become more mature.^[^ ^49^ ^]^ Recent research reported that cortical organoids reach postnatal stages approximately by day 300 of culture, as drawn by comparing the timeline of their neurodevelopment in culture to that of in vivo neurodevelopment, based on a comprehensive analysis of epigenetic and transcriptomic data.^[^ ^55^ ^]^ Our midbrain organoids were cultured for 77 days, and could similarly develop and mature over longer times to better reflect in vivo neurodevelopment. The cell type composition of our midbrain organoids was similar to that of the human fetal ventral midbrain at 8–10 weeks of in vivo embryonic development.

Once the midbrain organoids were established as a viable model for midbrain neurodevelopment, they were treated with fentanyl. Acute fentanyl treatment (74 nm for 4 hours) increased opioid receptor expression and dopamine release but did not elicit significant changes in gene expression profiles related to cell lineage development and neuron projection. In contrast, chronic fentanyl treatment (74 nm for 53 or 77 days) did affect the cell fate of iPSCs. Using single‐cell transcriptome profiling, we found that chronic fentanyl exposure impaired the neurodevelopment of midbrain organoids, as indicated by an increased neuroprogenitor cell pool and a delay in neuronal differentiation and maturation. Moreover, gene expression and pathways related to neural development were significantly altered in long‐term fentanyl‐treated organoids. While additional testing across various human iPSC lines is necessary to validate the effects of opioids, our current findings suggest that our midbrain organoids offer a promising model for investigating the impact of opioids on midbrain neurodevelopment.

The results are consistent with the clinical observation of impaired neurodevelopment in neonates with in utero chronic opioid exposure. Delayed embryonic neurodevelopment is associated with psychomotor development delays, lower IQ scores, and higher total behavioral problem scores in childhood.^[^ ^12^ ^]^ Neonates often suffer from withdrawal symptoms, including high‐pitched crying, poor feeding, exaggerated Moro reflex, irritability, and trouble sleeping.^[^ ^56^ ^]^ These symptoms are attributed to NAS and are exacerbated by the sudden discontinuation of opioids upon birth. Our results indicate that midbrain synaptic activity was reduced to a basal level with chronic fentanyl treatment, and neurodevelopment resumes in transcriptomes after 2 days of fentanyl withdrawal. However, in children, NAS symptoms persist by affecting the central and autonomic nervous systems, as well as the gastrointestinal system.^[^ ^57^ ^]^ The findings of this study may be useful in developing therapeutics to treat NAS and chronic in utero opioid exposure.

Challenges persist in achieving native brain functionality in a midbrain organoid model and in correlating organoid‐based findings with clinical readouts. Brain organoids lack full cell maturation, heterogeneity, architecture, vasculature, and the microenvironment of the human brain.^[^ ^30^, ^58^ ^]^ Further characterization of organoids is needed to better understand these limitations and enhance the model's fidelity. Advances in spatial multi‐omics, high‐throughput transcriptomics, and proteomics offer opportunities for characterizing the dynamics of brain neurogenesis and synaptogenesis in organoids.^[^ ^59^ ^]^ Future studies could validate these models through human studies or animal models.

Another limitation is the use of a single brain region‐specific organoid. Neuropsychiatric disorders, including substance abuse, affect multiple brain regions and involve complex neural circuits.^[^ ^60^ ^]^ While midbrain organoids provide valuable insights into opioid effects on the midbrain, they lack the functional neural circuits present in the native central nervous system and provide only a partial view. Comprehensive studies using various region‐specific organoids are necessary to obtain a more holistic understanding.

A final limitation is that brain organoids cannot easily reveal the effects of multiple drug (polydrug) abuse, as they cannot attribute specific downstream effects to individual drugs. To gain a mechanistic understanding of each drug's effects, they must be studied individually before examining combinations of drugs.

Long‐term opioid treatment induces extensive transcriptional changes related to neurodevelopment and neuronal communication in a midbrain organoid model. Future studies correlating clinical readouts with transcriptomic markers will allow a more comprehensive evaluation of drug abuse consequences and aid in identifying therapeutic targets.

## 4 Conclusion

In midbrain organoids, short treatment with fentanyl increased dopamine release but did not significantly change the expression of genes involved in cell lineage development. In contrast, chronic exposure to fentanyl impaired cell subtype specification and altered synaptic activities in neurons, indicating arrested neurodevelopment. Upon fentanyl withdrawal, neurodevelopment returned to normal at the transcriptomic level. Our study provides insights into the molecular mechanisms of opioid responses in both neural and non‐neural cells and identifies pathways that could be targeted to improve treatments for substance abuse and neonatal abstinence syndrome.

## 5 Experimental Section

### Culture of Human Induced Pluripotent Stem Cells (iPSCs)

The FA‐11 iPSC line was generously provided by the Columbia Stem Cell Initiative with an approved IRB. FA‐11 cells were derived from human dermal fibroblasts of a healthy donor.^[^ ^61^ ^]^ The iPSCs were maintained under feeder‐free conditions on Matrigel‐coated plates in mTeSR Plus media (Stemcell Technologies). The media was changed daily, and iPSC cultures were split 1:6–1:10 every 5 days using ReLeSR (Stemcell Technologies). All experiments were exclusively performed using the FA‐11 human iPSC line. The WTC human iPSC line was utilized solely to evaluate the consistency of organoid formation.

### Generation of Midbrain Organoids

iPSCs before passage 30 were used to generate organoids. To form embryoid bodies (EBs), iPSCs were dissociated into single cells with accutase (Gibco), and 8000 cells were plated in each well of untreated U‐bottom 96‐well culture plates (Corning) with mTeSR plus media and 10 µm ROCK inhibitor Y27632 (Tocris Bioscience). To induce iPSC differentiation toward a floor plate, EBs were treated with the dual‐SMAD inhibition factors LDN 193 189 and SB431542, and the Wnt pathway activator CHIR99021.^[^ ^62^ ^]^ For efficient midbrain patterning toward a ventral mesencepthalic fate, Sonic Hedgehog (SHH) signaling was activated with the small molecule agonist purmorphamine in combination with recombinant SHH^[^ ^63^ ^]^ (Figure 1a). Detailed product information is listed in Table S2 (Supporting Information).

On day 0, neuronal induction medium composed of 15% Knockout serum replacement (Gibco), 1% GlutaMax (Gibco), 1% minimum essential media‐nonessential amino acids (MEM‐NEAA) (Gibco), and 0.1% β‐mercaptoethanol (Gibco) in Knockout DMEM/F12 (Gibco) supplemented with 100 nm LDN193189 (Stemgent) and 10 µm SB431542 (Tocris Bioscience) was used. On day 2, we changed to the neuronal induction media supplemented with LDN193189, SB431542, 100 ng mL^−1^ SHH (R&D Systems), and 2 µm purmorphamine (Calbiochem). On day 4, 3 µm CHIR99021 (Stemgent) was added to the medium. From days 6–8, the basal media containing LDN193189 and CHIR99021 was gradually replaced to a differentiation media containing DMEM/F12: Neurobasal (1:1) (Gibco), N2 supplement (Gibco), B27 supplement without vitamin A (Gibco), 1% GlutaMAX, 1% MEM‐NEAA, 0.1% β‐mercaptoethanol and 1% penicillin‐streptomycin (Gibco). On day 9, the differentiation media was supplemented with CHIR99021, 10 ng mL^−1^ BDNF (Prospec), 0.2 mm ascorbic acid (Sigma), 20 ng mL^−1^ GDNF (Prospec), 0.2 mm dibutyryl‐cAMP (Calbiochem), 1 ng mL^−1^ TGF‐β3 (R&D Systems), and 10 µm DAPT (Tocris). From day 11, organoids were cultured in the same media without CHIR99021. The media was changed every 3–4 days. On day 21, the organoids were transferred into untreated 24‐well plates by using a 200 µL tip with a wide bore opening. Organoid growth was monitored by bright‐field microscopy (Nikon Eclipse) for 53 days, and the diameter was measured using Image J.

### Acute and Chronic Opioid Treatment

For acute opioid treatment, day 95 organoids were treated with fentanyl (FTY, Cerilliant, F‐002), oxycodone (OXC, Cerilliant, O‐002), or hydrocodone (HDC, Cerilliant, H‐003) at concentrations of 4 nm, 7 nm, 19 nm, 37 nm, and 74 nm for 4 h. Cytotoxicity was assessed with a CellTiter‐Glo Luminescent Cell Viability Assay (Promega). Organoids were lysed using CellTiter‐Glo reagent, mixed by shaking for 5 min, incubated for 25 min at room temperature, and recorded luminescent signal. Changes in mRNA expression of opioid receptors and dopamine release levels caused by opioid treatment were evaluated by qPCR and HPLC analysis, respectively. For chronic opioid treatment, fentanyl (74 nm in culture media) was used to treat organoids starting on day 1 of neuronal induction. The media was fully changed with fresh media containing fentanyl every 2 days. On day 77, to analyze the effects of opioid withdrawal, media was replaced with fresh media without fentanyl and organoids were incubated for 2 days prior to scRNA‐seq analysis.

### Immunohistochemistry

Organoids were fixed with 4% paraformaldehyde for 1 h, placed in a 30% sucrose solution in PBS overnight, and embedded in O.C.T. compound (Tissue‐Tek) for cryosectioning. Frozen organoids were cryosectioned at a thickness of 20 µm. The slides were heated to 95 °C in citrate buffer (10 mm sodium citrate, 0.05% Tween 20, pH 6.0) for antigen retrieval. For immunohistochemistry, slides of organoid cryosections were permeabilized with 0.2% TritonX‐100 in PBS for 20 min at room temperature, then blocked with 3% bovine serum albumin (BSA, Sigma) in DPBS containing 0.1% Triton X‐100 for 1 h at room temperature. The sections were incubated with primary antibodies overnight at 4 °C and with secondary antibodies for 1 h at room temperature (Table S3, Supporting Information). All sections were stained for cell nuclei with DAPI (Sigma). Images were acquired using a confocal microscope (LSM 710, Zeiss).

### Real‐Time PCR (qPCR)

Gene expression profiles of organoids were evaluated for 35 days by qPCR. Total RNA was isolated from organoids using TRIzol (Invitrogen) and was reverse‐transcribed using an iScript cDNA synthesis kit (Bio‐Rad) to produce cDNA. Quantitative qPCR was performed using PowerUp SYBR Green Master mix (ThermoFisher) and a QuantStudio 3 Real‐Time PCR System (Applied Biosystem). The ∆∆Ct method was used to normalize expression levels of each gene to those of GAPDH. Primer sequences are listed in Table S4(Supporting Information).

### Electrophysiology

Whole‐cell patch clamp recordings were taken from organoids as described previously.^[^ ^64^ ^]^ Day 35 organoids were attached to Matrigel‐coated cover glasses overnight. Recordings were performed at 22 °C under continuous perfusion (1 mL min^−1^) with a bath solution containing: 119 mm NaCl, 5 mm KCl, 30 mm HEPES, 2 mm MgCl_2_, 2 mm CaCl_2_, and 10 mm glucose. The solution was adjusted to 310 mOsm with sucrose and to pH 7.3 with KOH. Borosilicate glass pipettes with a tip resistance of 3–4 MΩ (G150F‐4, Warner Instruments) were pulled on a P‐97 Flaming‐Brown micropipette puller (Sutter Instruments) and filled with an internal solution containing 130 mm K‐gluconate, 10 mm KCl, 2 mm Mg‐ATP, 0.2 mm Li‐GTP, 0.6 mm CaCl_2_, 5 mm MgCl_2_, 0.6 mm EGTA, and 5 mm HEPES titrated to pH 7.1 and an osmolality of 310. Neurons were visualized under a 40X water immersion objective by DIC (Olympus). Recordings were performed with an Axopatch 700B amplifier (Molecular Devices) and digitized at 10 kHz with an ITC‐18 instrument (HEKA Instruments). Data analysis was performed using Clampfit 10 software (Molecular Devices, Sunnyvale, CA) and Matlab 8.0 (MathWorks, Natick, MA). In each neuron, input resistance, resting membrane potential, and spontaneous firing frequencies were monitored throughout the recording, and only cells with neuronal morphology and a stable baseline activity that continued for over 5 min were counted as tonically active. To determine the identity of recorded neurons, neurons were labeled with biocytin (Sigma–Aldrich) added to the patch pipette saline for at least 10 min, fixed in 4% PFA for 1 h, and immunostained with anti‐TH antibodies.

### Calcium Imaging

On day 180 of culture, fentanyl‐treated and untreated organoids were used for calcium imaging. The organoids were incubated with the cell‐permeable calcium indicator Fluo‐4 AM (ThermoFisher) for 30 min at 37 °C. Time‐lapse changes in Ca^2+^ levels in live organoids were imaged using a fluorescence microscope (Eclipse TS100, Nikon). To observe the instantaneous effect of fentanyl treatment on calcium channels, untreated organoids were recorded before and after the addition of fentanyl (74 nm). Organoids were imaged every 2 s at room temperature. The fluorescence intensity was determined by Image J and the data were normalized to ∆*F*/*F*_0_ using the equation y = (F_∆sec_ – F_0sec_)/F_0sec_.

### Dopamine Release Measurement

Intracellular and extracellular dopamine production in the organoids was characterized by HPLC. Three organoids per condition were transferred to a 96‐well plate on day 53. L‐Dopa (100 µm) was added to each well and incubated for 1–2 h. For extracellular dopamine measurements, the media was removed and the organoids were washed with PBS, followed by the immediate addition of Tyrode's containing KCl (40 mm). After 20–30 min of incubation, the sample in each well was collected and mixed with 0.12 m perchloric acid at a 1:1 (v/v) ratio. For intracellular dopamine measurements, all media was replaced with Tyrode's containing KCl and organoids were then dissociated. The lysate was mixed with 0.12 m perchloric acid at a 1:1 (v/v) ratio. After a 10–15 min incubation, cell debris was removed by centrifugation at 10 000 g and 4 °C and the supernatant was collected for HPLC. Organoid dopamine levels were determined by HPLC with electrochemical detection as described previously.^[^ ^65^ ^]^ Briefly, samples were separated on a VeloSep RP‐18, 3 µm, 100 mm x 3.2 mm column (PerkinElmer, Waltham, MA) with a Gilson 307 HPLC piston pump set to a flow rate of 0.7 mL min^−1^ and a mobile phase containing 45 mm NaH_2_PO_4_, 0.2 mm EDTA, 1.4 mm HSA, 5% methanol, pH 3.2. Dopamine was detected on an ESA Coulochem II electrochemical detector at 350 mV oxidation potential. Data were collected using Igor software, and dopamine concentration was calculated from areas under the HPLC peaks using calibration curves.

### Single‐Cell RNA Sequencing

Five independent samples of midbrain organoids were gently dissociated to single cells with papain enzymatic dissociation (Worthington Biochemical Corporation). After filtering cell clumps with a cell strainer (35 µm mesh), suspended single cells were encapsulated with barcoded beads in oil droplets by 10X Genomics Chromium technology. The resulting single cell 3′‐end libraries were sequenced on an Illumina NovaSeq 6000 Sequencing System (2 × 100 bp pair‐end) at the Single Cell Analysis Core of the Columbia Genome Center. 10X genomics' Cellranger pipeline v3.1.0 with human reference transcriptome GRCh38 was used to process the data. Details of the software and algorithms are provided in Table S5 (Supporting Information).

### Cell Type Annotations

The Seurat package (V3.0) in R (V3.6.3) was used to normalize the expression matrix and identify differentially expressed genes. The Glmnet package (V4.0) was used to assign cell types based on cells’ likelihood to an annotated human fetal midbrain dataset.^[^ ^36^, ^66^, ^67^ ^]^ First, both the experimental and reference datasets’ gene counts were transformed separately using the sctransform function in Seurat. Then Seurat's CCA integration method was used to account for technical differences between datasets, and the datasets in UMAP were co‐clustered.^[^ ^44^ ^]^ Then a multinomial logistic regression classifier (Glmnet R package) on the reference's integrated data excluding the “unknown” (Unk) cell type was trained and the cell type of both datasets was predicted. In non‐Unk reference cells, the classifier's accuracy was 99%. To validate the cell types assigned, the gene signatures of each cell type between each dataset were compared using highly correlated or anti‐correlated genes to predict the cell types in samples. These results showed similar gene patterns compared to the reference dataset. It was found that the cell type composition of the reference dataset and the dataset were similar, with some variation in percentages of progenitor cells, neuroblasts, and oligodendrocyte progenitor cells (OPCs) (Figure 1f; Figure S1e–h, Supporting Information). In the reference dataset, progenitors referred to the cells at the floor plate in a 6–11 weeks old human midbrain, with a high expression of *HMGA1*, *HMGB2*, *OTX2*, *FOXA2*, and *DMBX1*. Neuronal progenitors were cells expressing pro‐neurogenic genes such as *NEUROG1*, *NEUROD1*, *NEUROD4*, *NEUROG2*, and *NHLH1*. Sub‐clusters of the same cell type were grouped into one major type. For example, any cell type with hDA in the name was mapped to the DA major type; any cell type with Prog in the name was mapped to the Prog major type; any cell type with hNb in the name was mapped to the Nb major type. A detailed table of cell metadata table was deposited in Gene Expression Omnibus (GEO) under the accession number GSE260711.

### Differential Expression Analysis

Differentially expressed genes between samples or cell types were identified by using the FindMarkers function in Seurat. The following parameters were used: min.pct = 0.25, logfc.threshold = log(2). The top genes in each comparison group were selected with a pre‐filtering step (p_val_adj < 1e‐10, pct.1 > 0.1, pct.2 > 0.1, avg_logFC>5 or ←5) to remove low count and insignificant genes, then were ranked by the absolute value of the log fold change. A heatmap of top differentially expressed genes is shown in Figure S2c,d (Supporting Information).

### Single Sample Gene Set Enrichment Analysis (ssGSEA)

ssGSEA was performed to identify activated pathways in samples using a pipeline previously described for identifying subtypes with enriched gene sets.^[^ ^68^ ^]^ Briefly, a normalized gene expression matrix (generated by Seurat sctransform function) and gene sets of interest (from GSEA Molecular Signatures Database v7.2 and Gene Ontology Database) were used as inputs. Enrichment scores of a certain gene set were calculated for both the experimental dataset and a random permutated dataset of 1000 cells. *p*‐values of each gene set were computed by comparing the dataset to the permutated dataset for each single cell. Then the –log_10_(*p*‐values) was plotted out showing the enriched gene sets grouped by cell type or treatment using the ComplexHeatmap R package (V2.5.6)^[^ ^69^ ^]^ (Figures 4i and 5c).

### Construction of the Lineage Trajectory

Monocle3 (V0.2.0) and scVelo (V0.2.1) were used to identify the cell fate decision genes and pseudotime from the expression matrix.^[^ ^43^ ^]^ Monocle3 first projected cells onto a low‐dimensional space using UMAP,^[^ ^44^ ^]^ then grouped similar cells using the Louvain community detection algorithm and constructed trajectories based on the divergence or convergence of cell types (Figure 3f–g). For RNA velocity, first, a loom file of spliced and unspliced mRNAs was created from an annotated sequencing bam file using the Velocyto package (V0.17.16) in Python (V3.6).^[^ ^70^ ^]^ Then calculations of cell dynamics were performed using the scVelo package (V0.2.1) in Python (V3.6)^[^ ^32^ ^]^ (Figures 3h–k and 5b). The scVelo algorithm is based on RNA velocity, requiring minimal prior knowledge about the sample molecular signatures. It solves the full transcriptional dynamics and has been effectively tested in dissecting kinetics in neurogenesis.

### Identification of Gene Regulatory Network

The Scenic package (V1.2.2) in R (V3.6.3) was used with default settings to identify key cell fate decision transcription networks.^[^ ^71^ ^]^ Briefly, sets of genes that were co‐expressed with transcription factors were identified using the GENIE3 module. The top five transcription factors for each target gene were kept. Then, the RcisTarget module sought out the enriched cis‐regulatory motifs of candidate transcription factors and predicted the candidate target genes. Lastly, the AUCell algorithm in the SCENIC package was used to score the activity of each regulon in each cell. The scores were grouped by cell types and their RegulonAUC values in the heatmaps were plotted to show the regulon activities of master regulators (Figures 4a–g and 5a; Figure S5, Supporting Information). This method is robust against normalization methods and cell dropouts, as consistent results were obtained after testing multiple runs of the same datasets under different normalization pipelines.

### Sequencing Data Availability

The accession number for the single‐cell data reported in this study is GEO: GSE260711.

## Conflict of Interest

The authors declare no conflict of interest.

## Supporting information

Supporting Information

Supplemental Movie 1

Supplemental Movie 2

## Data Availability

The data that support the findings of this study are available in the Gene Expression Omnibus database with accession number GSE260711.
