# Supplementary material for: Chronic Opioid Treatment Arrests Neurodevelopment and Alters Synaptic Activity in Human Midbrain Organoids
Source: Adv Sci (Weinh). 2024 Mar 28;11(21):2400847. doi: 10.1002/advs.202400847 (PMC11151039; doi:10.1002/advs.202400847)
Supplement: Supplementary file 1 — Supporting Information [file ADVS-11-2400847-s003.pdf]

ADVANCED SCIENCE
Open Access

Supporting Information

for *Adv. Sci.*, DOI 10.1002/advs.202400847

Chronic Opioid Treatment Arrests Neurodevelopment and Alters Synaptic Activity in Human Midbrain Organoids

Hye Sung Kim, Yang Xiao, Xuejing Chen, Siyu He, Jongwon Im, Moshe J. Willner, Michael O. Finlayson, Cong Xu, Huixiang Zhu, Se Joon Choi, Eugene V. Mosharov, Hae-Won Kim, Bin Xu and Kam W. Leong**

Supporting Information

Chronic opioid treatment arrests neurodevelopment and alters synaptic activity in human midbrain organoids

Hye Sung Kim, Yang Xiao, Xuejing Chen, Siyu He, Jongwon Im, Moshe J. Willner, Michael O. Finlayson, Cong Xu, Huixiang Zhu, Se Joon Choi, Eugene V. Mosharov, Hae-Won Kim, Bin Xu, and Kam W. Leong**

Figure S1

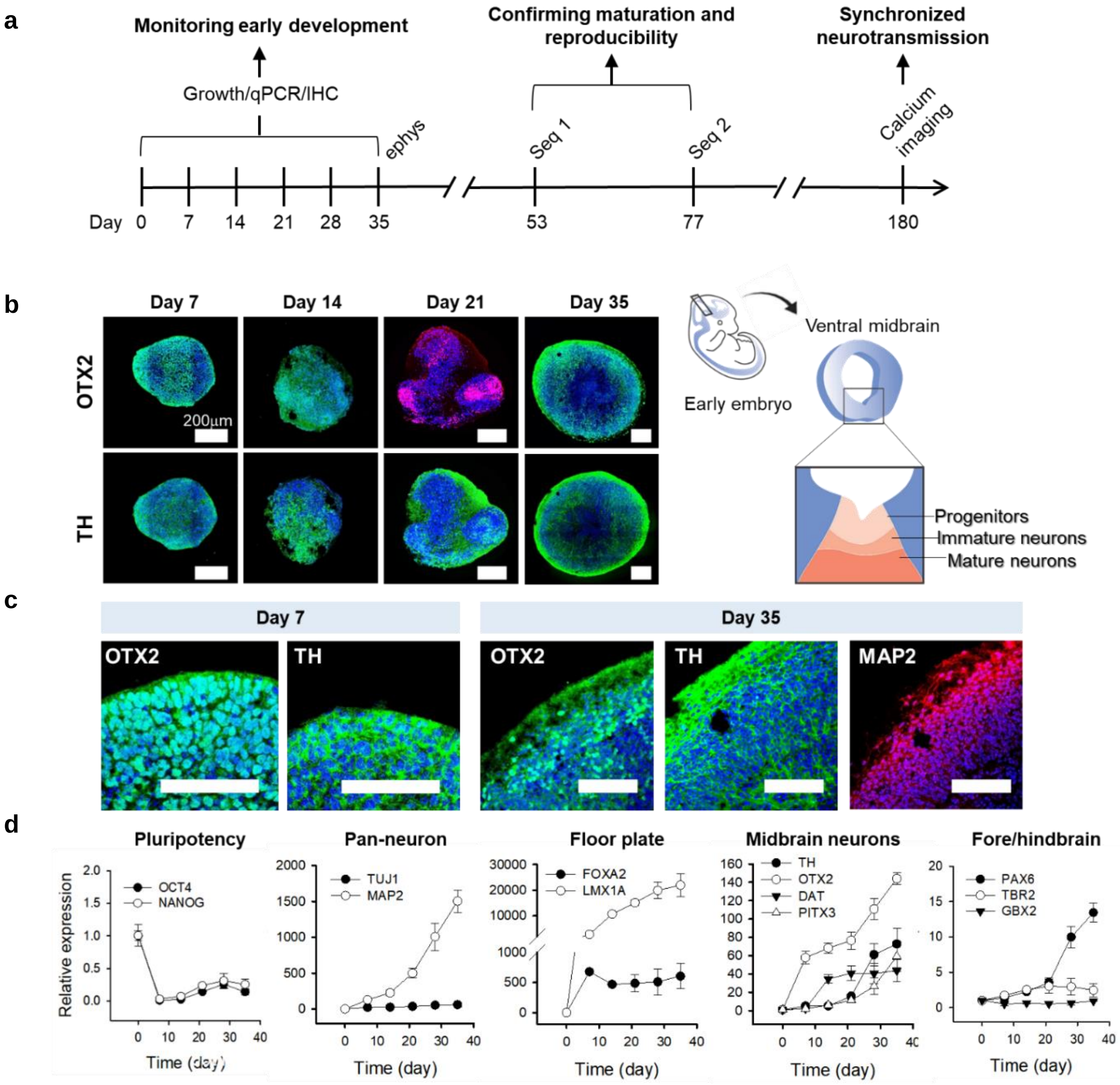

Figure S1 (cont'd)

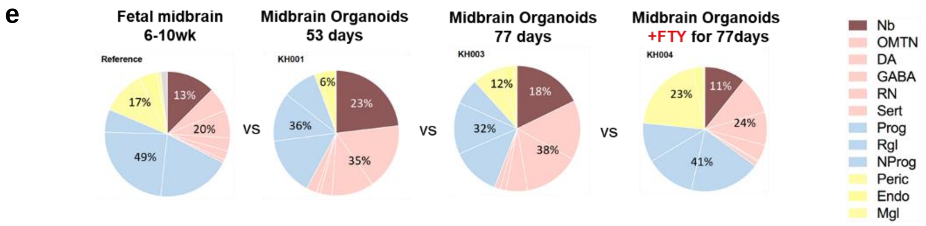

Sample	Sample ID	Progenitors				Neurons					Non-neurons			
		Nb	Prog	Rgl	NProg	OMTN	DA	GABA	RN	Sert	Peric	Endo	Mgl	OPC
53d UT	KH001	23.09%	15.13%	12.65%	8.75%	17.14%	10.83%	3.06%	0.95%	2.70%	1.42%	4.01%	0.26%	0.00%
77d UT	KH003	17.76%	12.54%	13.35%	6.64%	15.34%	14.26%	5.49%	1.48%	1.52%	7.75%	2.83%	1.04%	0.00%
77d FTY	KH004	10.71%	18.49%	12.92%	10.15%	9.85%	8.58%	4.21%	0.40%	1.27%	20.16%	2.54%	0.73%	0.00%
Ref	Ref	12.51%	19.06%	23.78%	5.90%	6.25%	7.20%	3.48%	2.36%	0.83%	11.27%	4.54%	1.12%	1.71%

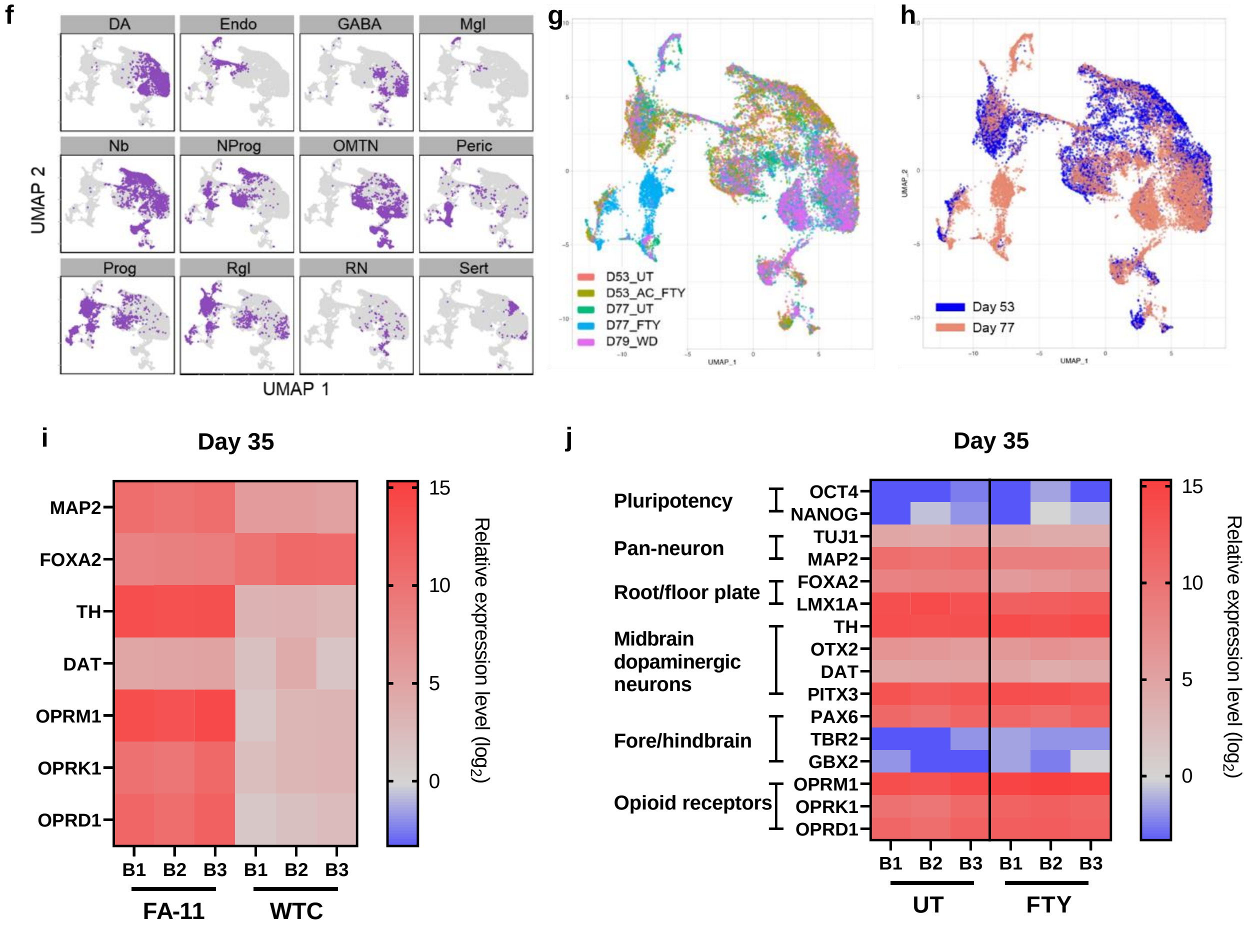

Figure S1. Midbrain-regional development and cell type identification and composition of iPSC-derived midbrain-like organoids by scRNA-seq, related to Figure 1. (a) A timeline of assays. (b-c) Immunocytochemical staining of organoids for OTX2, TH, and MAP2. The unique zonal cytoarchitecture was clearly shown starting on day 21 of culture. (d) Gene expression profiles of day 35 organoids by qPCR (n=3-5 organoids, three independent experiments). (e-h) The analysis was based on comparison to a dataset of human fetal ventral midbrain of *in vivo* embryonic development at 6-10 weeks. (e) Cell type compositions of midbrain organoids at days 53 and 77 of *in vitro* differentiation (Supplementary to Figure 1G). (f) Cell types shown as monochrome plots in UMAP (supplementary to Figure 1f). (g) Distribution in UMAP by sample (supplementary to Figure 1g). (h) Distribution in UMAP by organoid age (supplementary to Figure 1g). (i-j) Heatmap of qPCR data showing the relative expression of day 35 organoids (i) in two iPSC lines, FA-11 and WTC (n=3-7 organoids,

three independent batches), and (j) from three independent batches of UT and FTY made from FA-11.

Figure S2

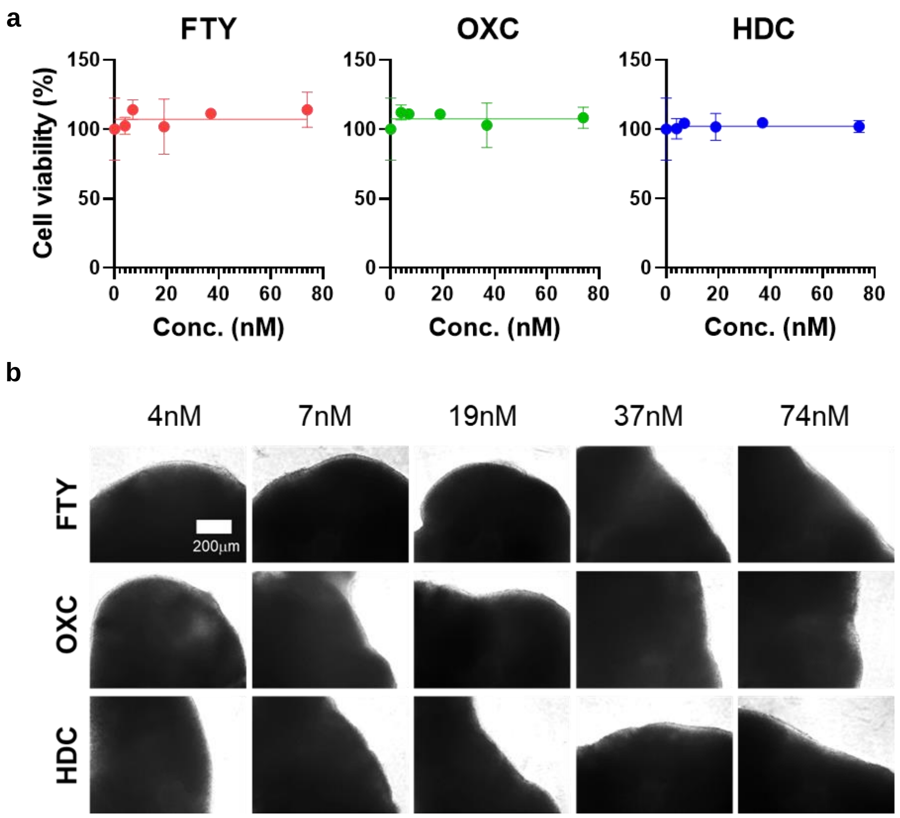
a
FTY
OXC
HDC
Cell viability (%)
150
100
50
0
0
20
40
60
80
Conc. (nM)
b
4nM
7nM
19nM
37nM
74nM
FTY
OXC
HDC
200µm

Figure S2 (cont'd)

c

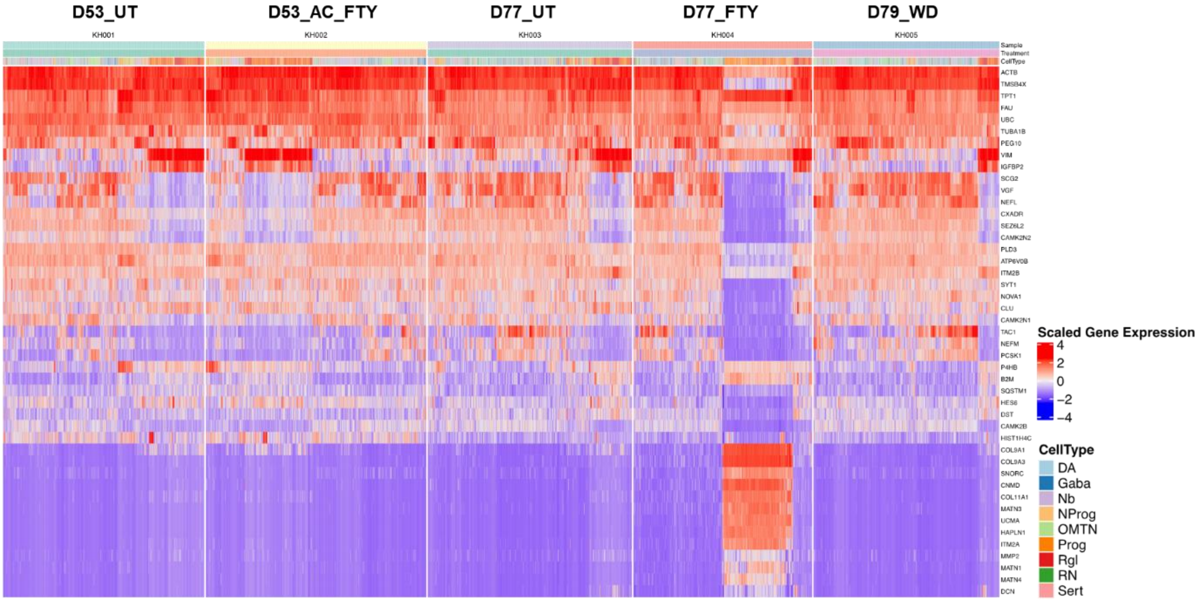

d

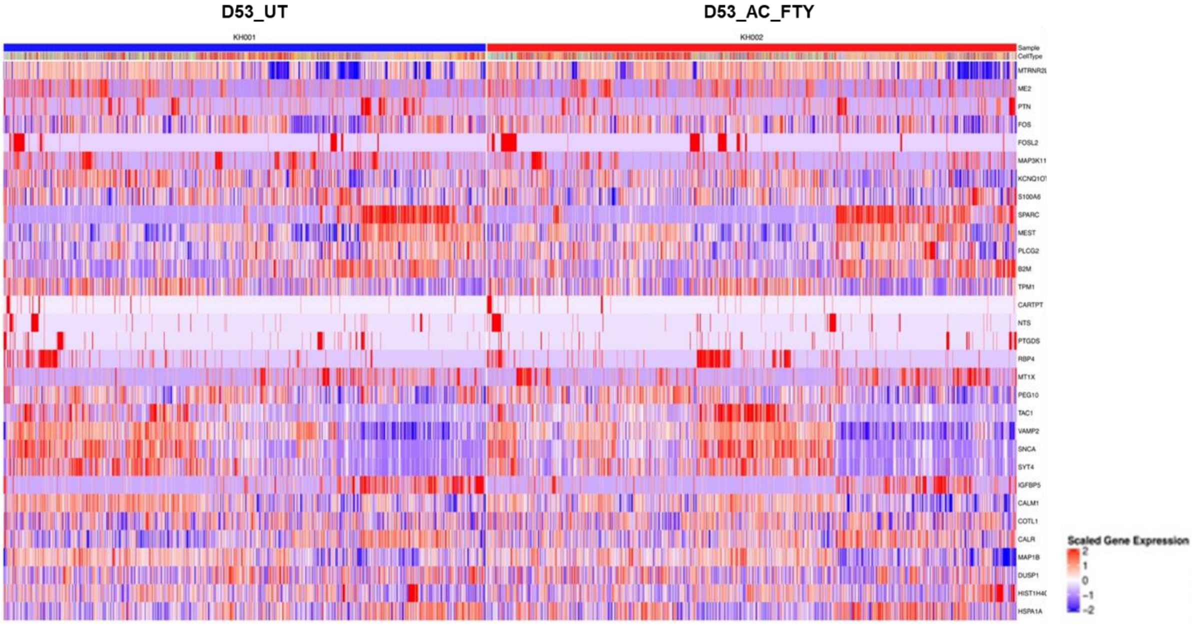

Figure S2. Cell viability tests of organoids with various treatments and differentially expressed genes, related to Figure 2. (a) Cell viability depending on opioid concentration (n=3 organoids, day 90 organoids, two independent experiments). (b) Bright-field images showing no noticeable dissociation of organoids. (c) Top differentially expressed genes among all samples. (d) Top differentially expressed genes detected in acute fentanyl treatment (comparing D53_UT vs D53_AC_FTY).

Figure S3

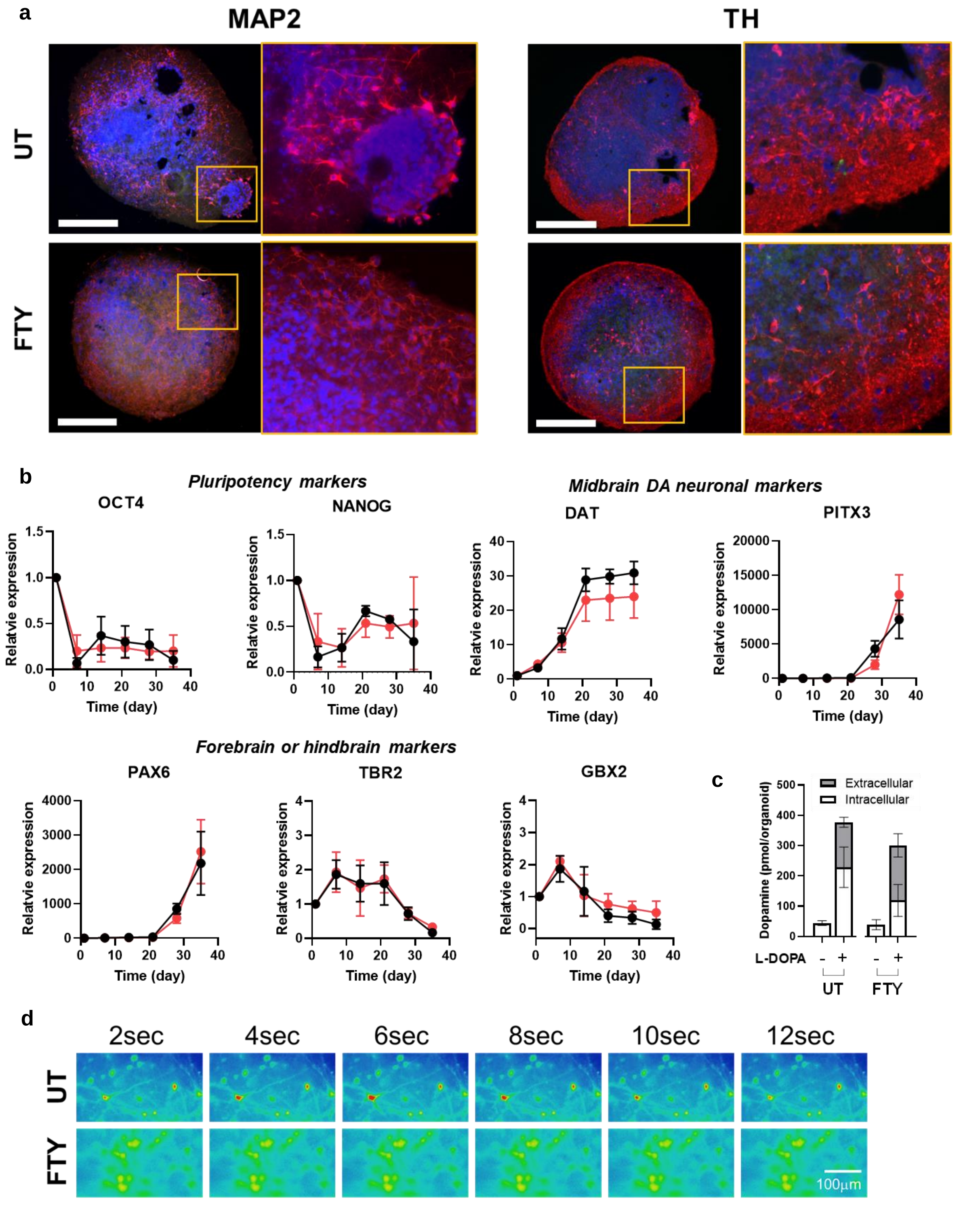
a
MAP2
TH
UT
FTY
b
Pluripotency markers
OCT4
NANOG
Midbrain DA neuronal markers
DAT
PITX3
Relatvie expression
Time (day)
Forebrain or hindbrain markers
PAX6
TBR2
GBX2
c
Extracellular
Intracellular
Dopamine (pmol/organoid)
L-DOPA
UT
FTY
d
2sec
4sec
6sec
8sec
10sec
12sec
100µm

Figure S3 (cont'd)

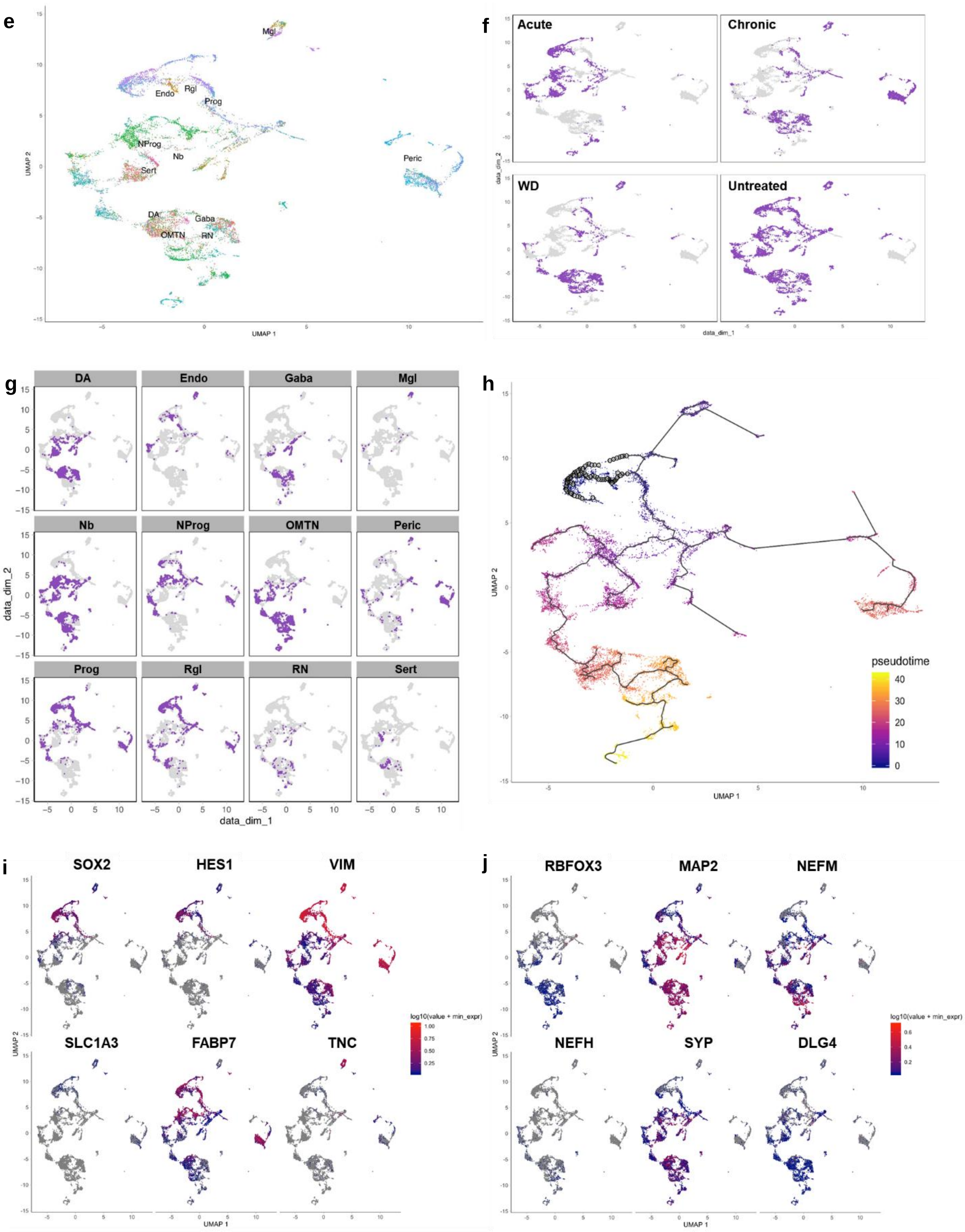

Figure S3 (cont'd)

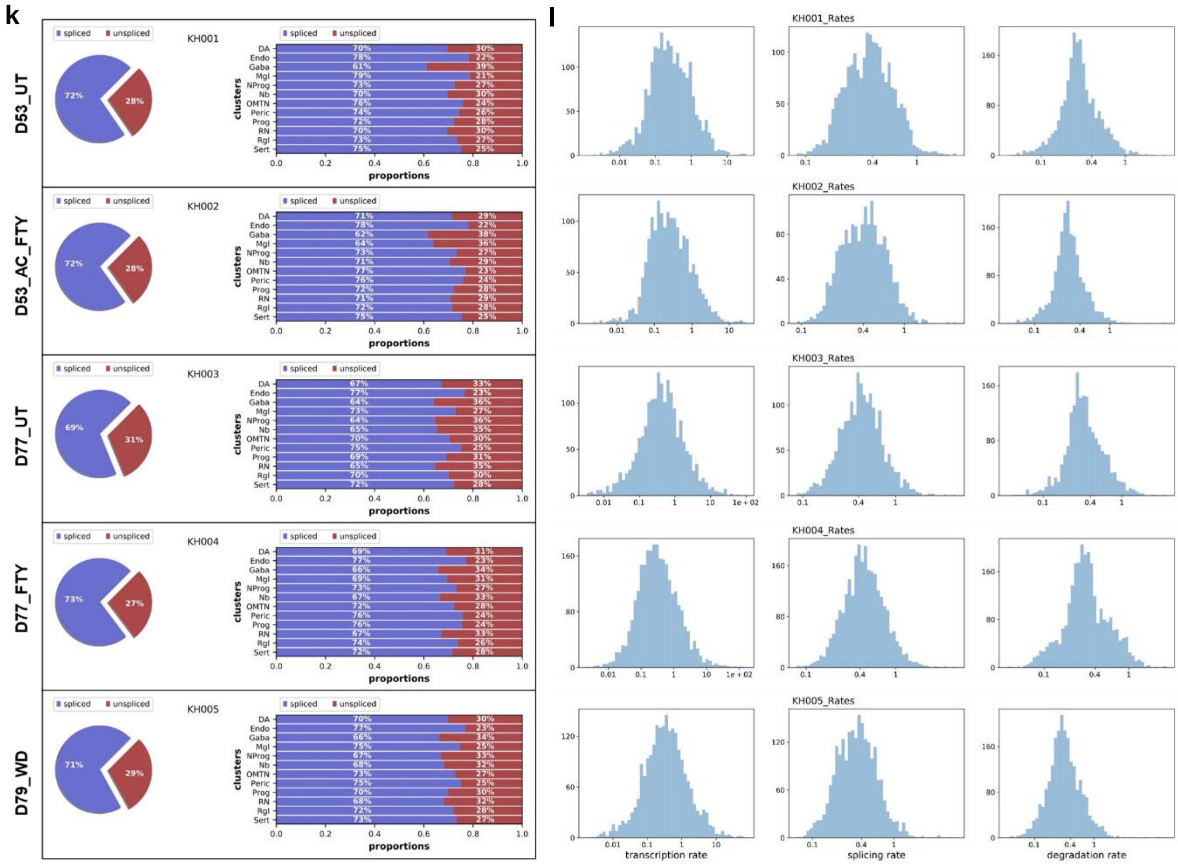

Figure S3 (cont'd)

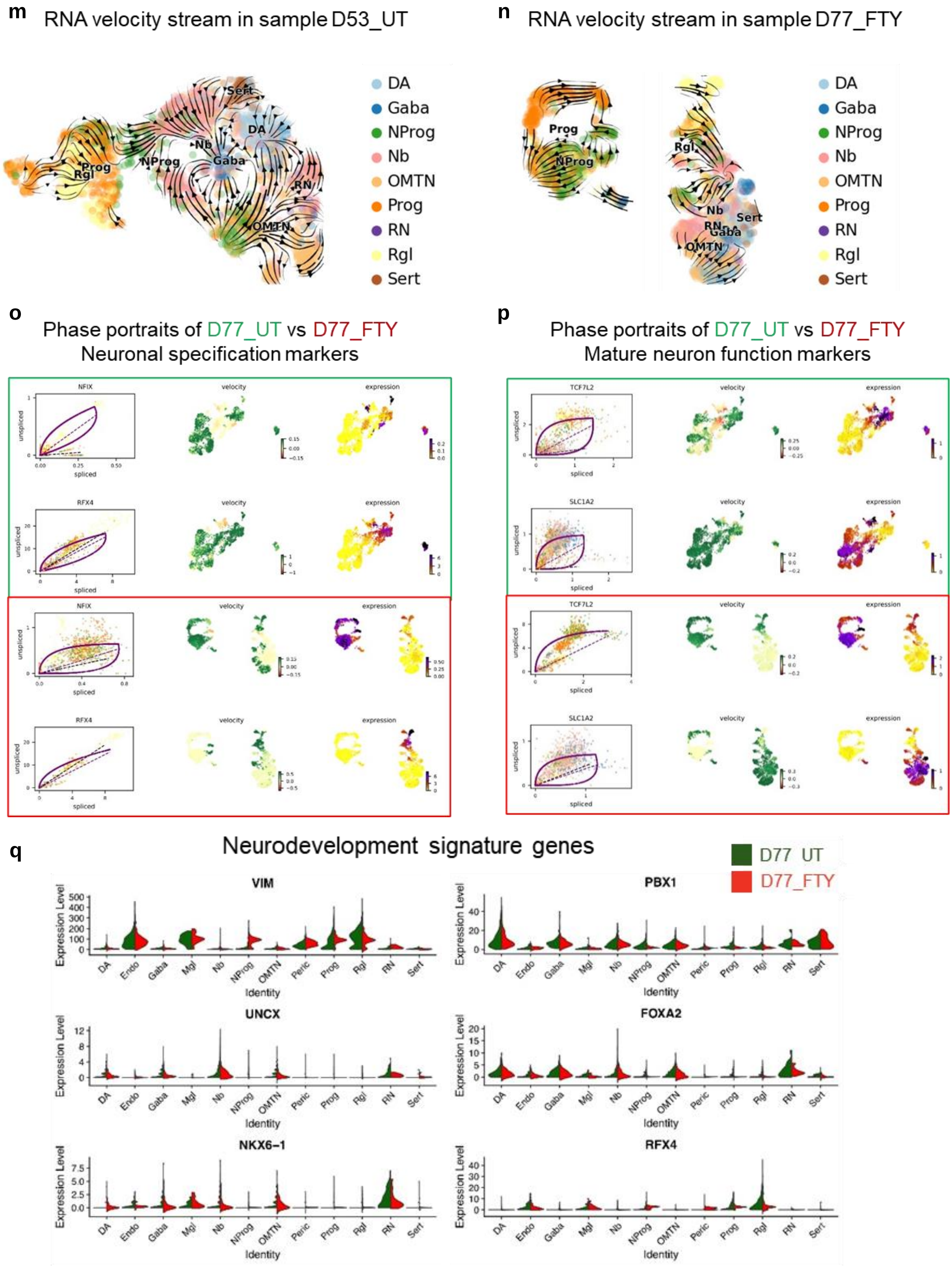

Figure S3. Effects of chronic opioid exposure on midbrain organoid development, related to Figure 3. (a) Immunocytochemical staining of organoids for MAP2 and TH in day 134 organoids. (b) Fold-changes of pluripotency genes (*OCT4* and *NANOG*), midbrain dopaminergic neuronal genes (*DAT* and *PITX3*), and forebrain or hindbrain genes (*PAX6*,

TBR2, and *GBX2*) (n=5-7 organoids, three independent experiments). (c) Dopamine synthesis and release of organoids with or without chronic fentanyl treatment (n=3 organoids, day 90, three independent experiments). (d) Calcium imaging of day 180 organoids. Organoids with chronic fentanyl treatment showed no calcium influx (Movie S2). (d-i) Cell lineage trajectory analysis (Monocle). (e) Monocle plot showing the distribution of all samples by cell type. (f) Monochrome plots showing the distribution of each sample. (g) Monochrome plots showing each cell type. (h) Lineage trajectory in pseudotime. (i) Feature plots of stem cell markers (*SOX2*, *HES1*, *VIM*, *SLC1A3*, *FABP7*, and *TNC*). (j) Feature plots of mature neuron markers (*RBFOX3*, *MAP2*, *NEFM*, *NEFH*, *SYP*, and *DLG4*). (j-k) Detailed plots of cell lineage trajectory analysis by RNA velocity. (k) Proportions of spliced and unsliced mRNAs for all samples. (l) Dynamic rates of RNA velocity in all five samples. (m-q) Chronic fentanyl treatment impairs neurodevelopment, shown by RNA velocity. (m, n) RNA velocity computed based on spliced / unspliced mRNAs in D53_UT and D77_FTY sample. (o, p) Phase portraits of key genes in neuronal specification or mature neuron function. (q) Violin plots of signature genes in neurodevelopment.

Figure S4

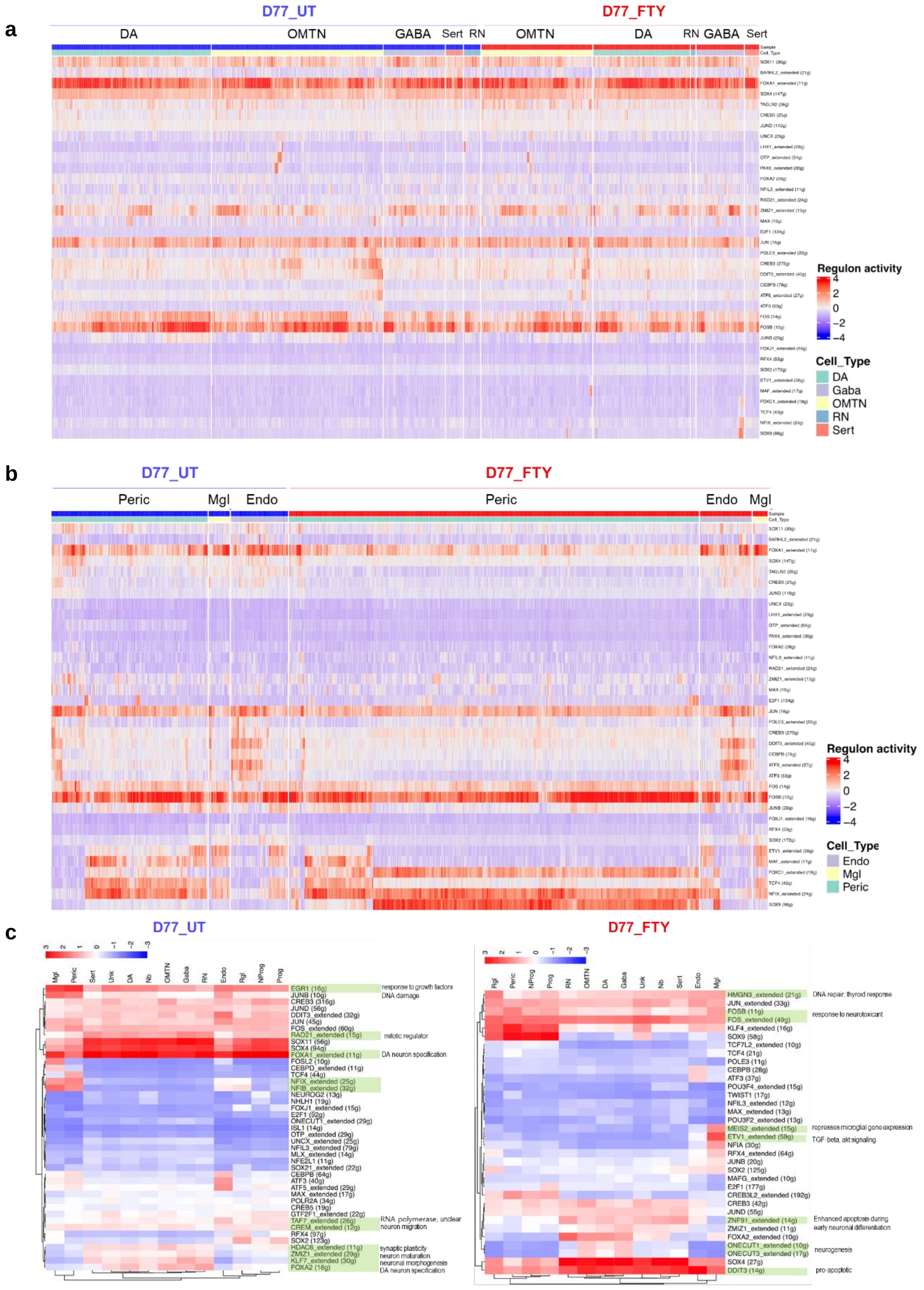
a
D77_UT
D77_FTY
DA
OMTN
GABA
Sert
RN
Regulon activity
Cell_Type
DA
Gaba
OMTN
RN
Sert
b
D77_UT
D77_FTY
Peric
Mgl
Endo
Regulon activity
Cell_Type
Endo
Mgl
Peric
c
D77_UT
D77_FTY
response to growth factors
DNA damage
mitotic regulator
DA neuron specification
RNA polymerase, unclear
neuron migration
synaptic plasticity
neuron maturation
neuronal morphogenesis
DA neuron specification
DNA repair, thyroid response
response to neurotoxicant
represses microglial gene expression
TGF-beta, akt signaling
Enhanced apoptosis during early neuronal differentiation
neurogenesis
pro-apoptotic

Figure S4 (cont'd)

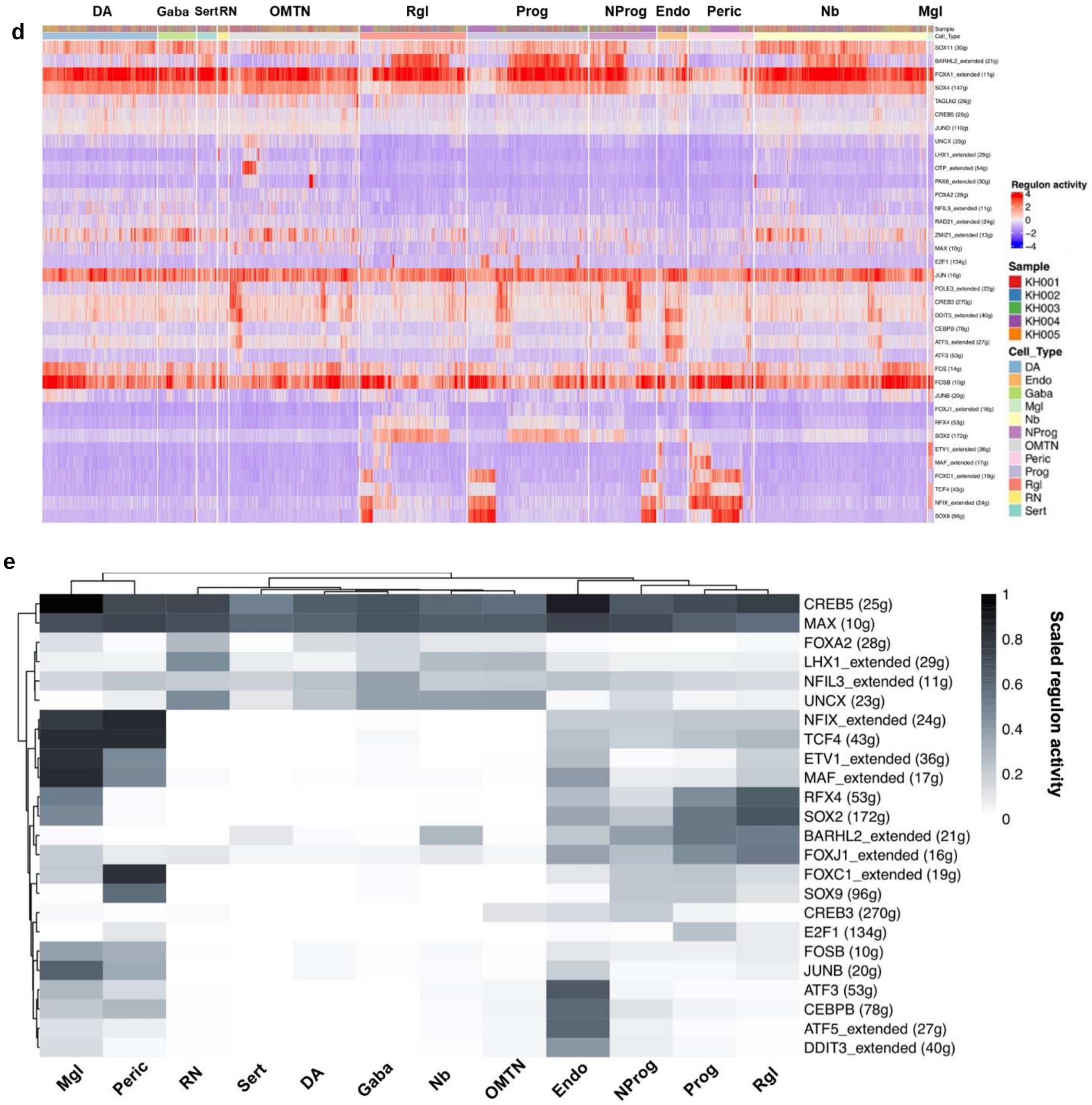

Figure S4 (cont'd)

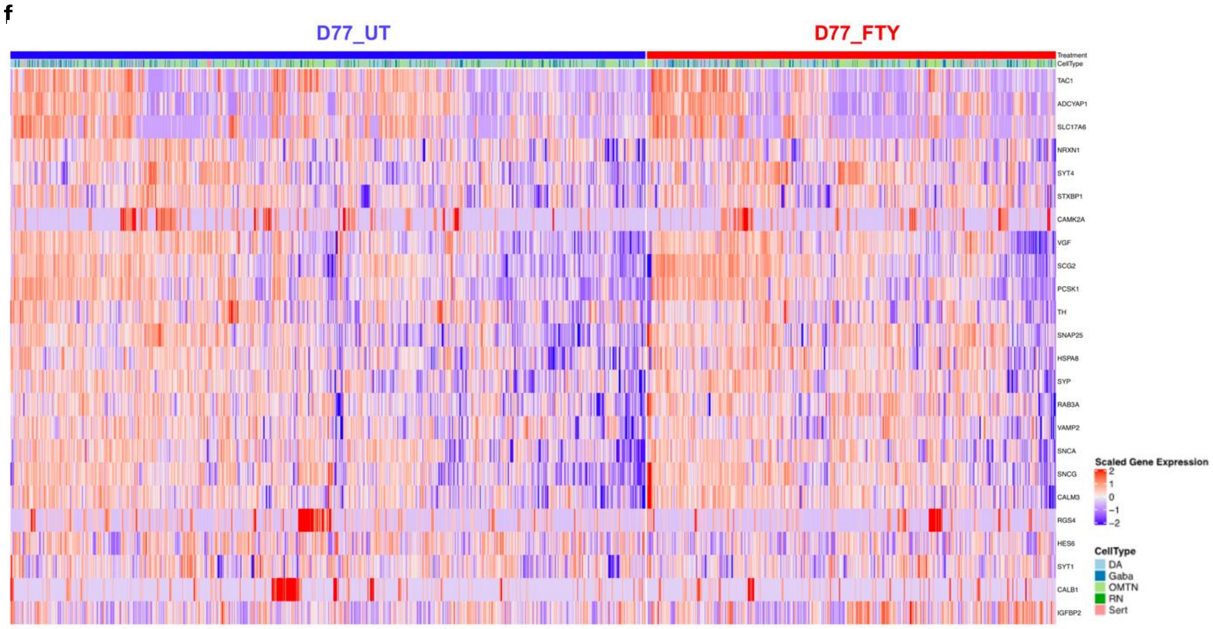

Figure S4 (cont'd)

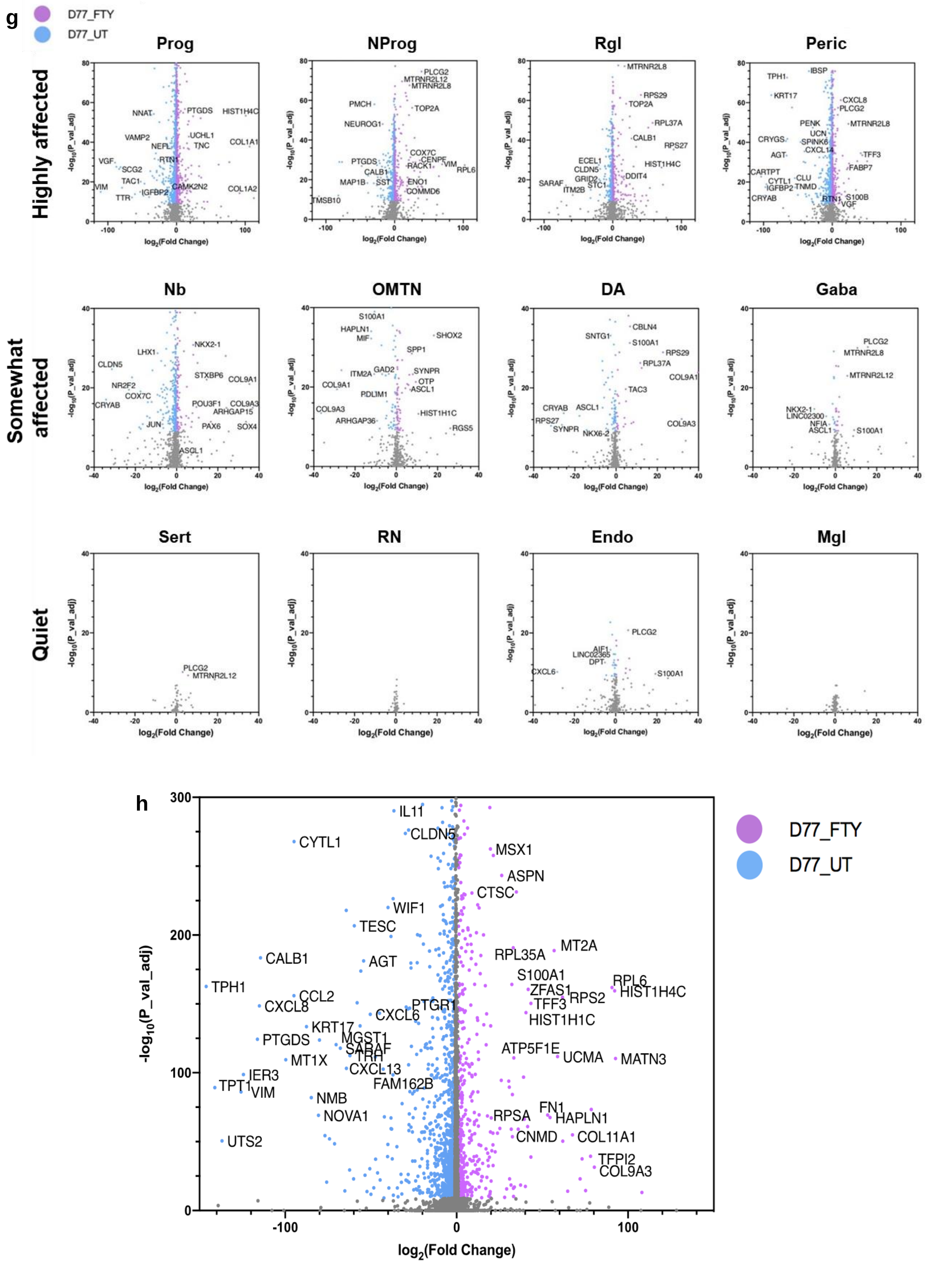

Figure S4 (cont'd)

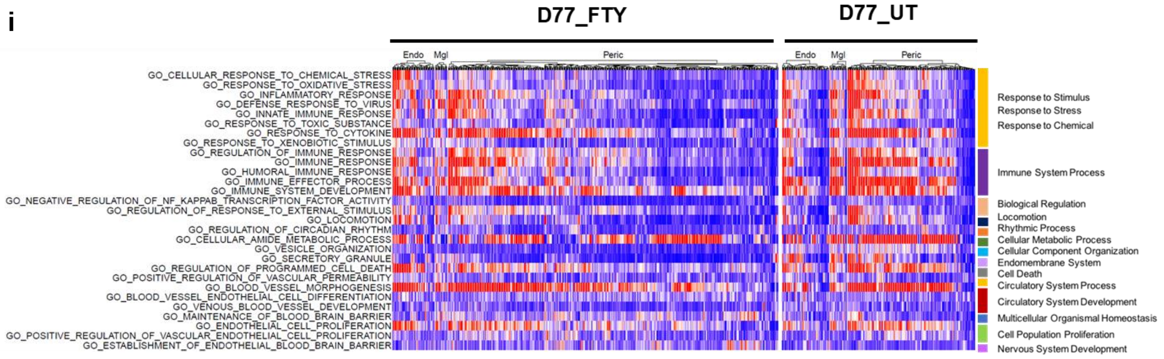

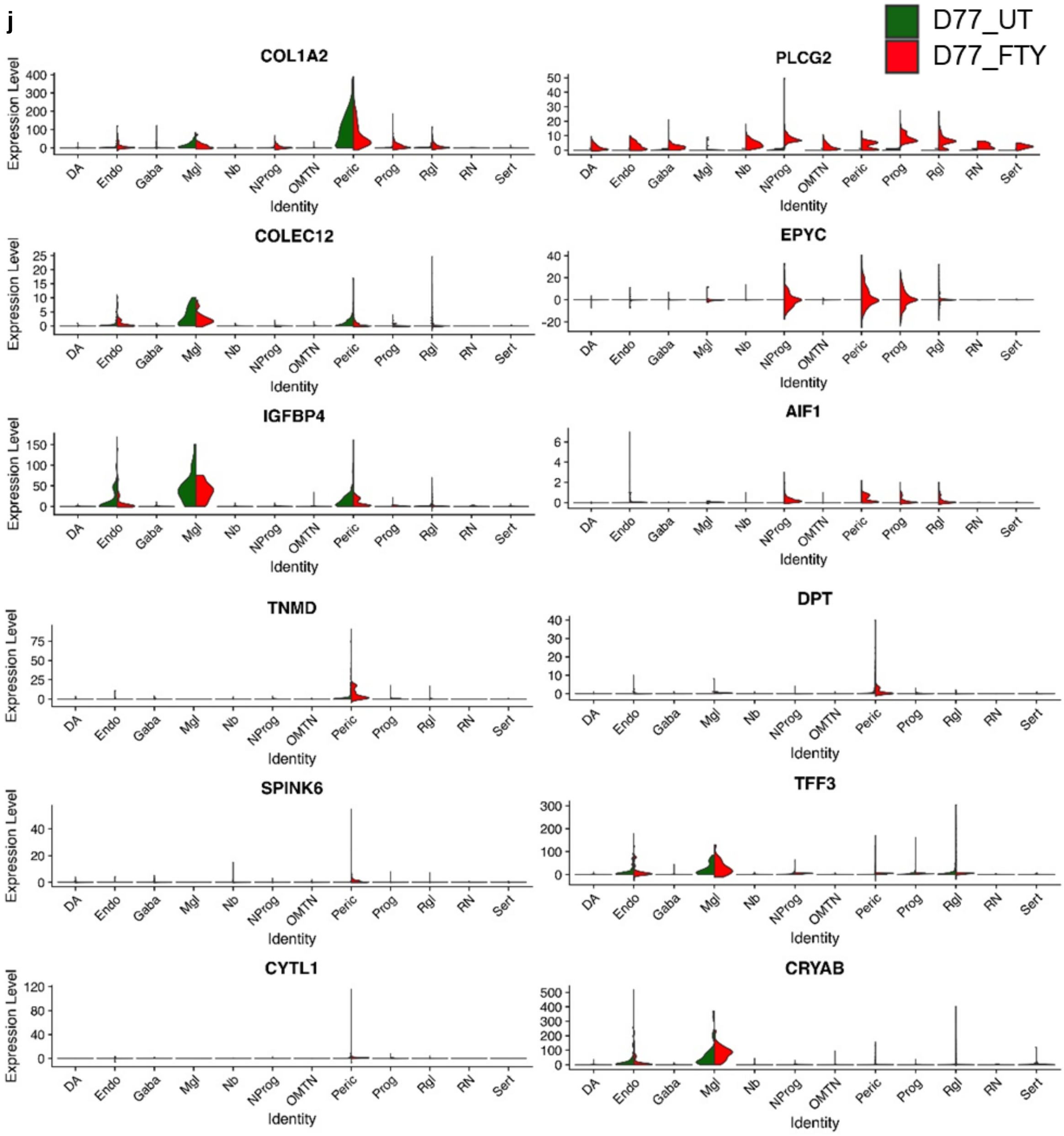

Figure S4. Cell-type specific response to chronic fentanyl treatment (KH004 vs. KH003), related to Figure 4. (a-c) Critical Regulators of cell identity, constructed using SCENIC R. (a) Master regulators identified in each single neuron. Regulon activity is colored by scaled AUCell scores. (b) Master regulators identified in each mesoderm-derived cells. (c) Master

regulators identified in sample. (d-e) Activities of gene regulatory network in all samples. (d) Master regulators identified in each single cell. Regulon activity is colored by scaled AUCell scores. (e) Binarized regulon activity matrix by applying a cutoff minimum of 0.2 in AUCell score. (f) Differentially expressed genes in neurons in response to chronic fentanyl treatment. (g-h) Differential expression analysis showing cell-type specific response to chronic fentanyl treatment. (g) Differential expression by cell type. (h) Volcano plots of all cells in each sample. (i-j) Cell-type specific response to chronic fentanyl treatment. (i) Mesoderm-derived cells; stimulus response and metabolic process. (j) Comparison of marker gene profiles in mesoderm-derived cells.

Figure S5

a

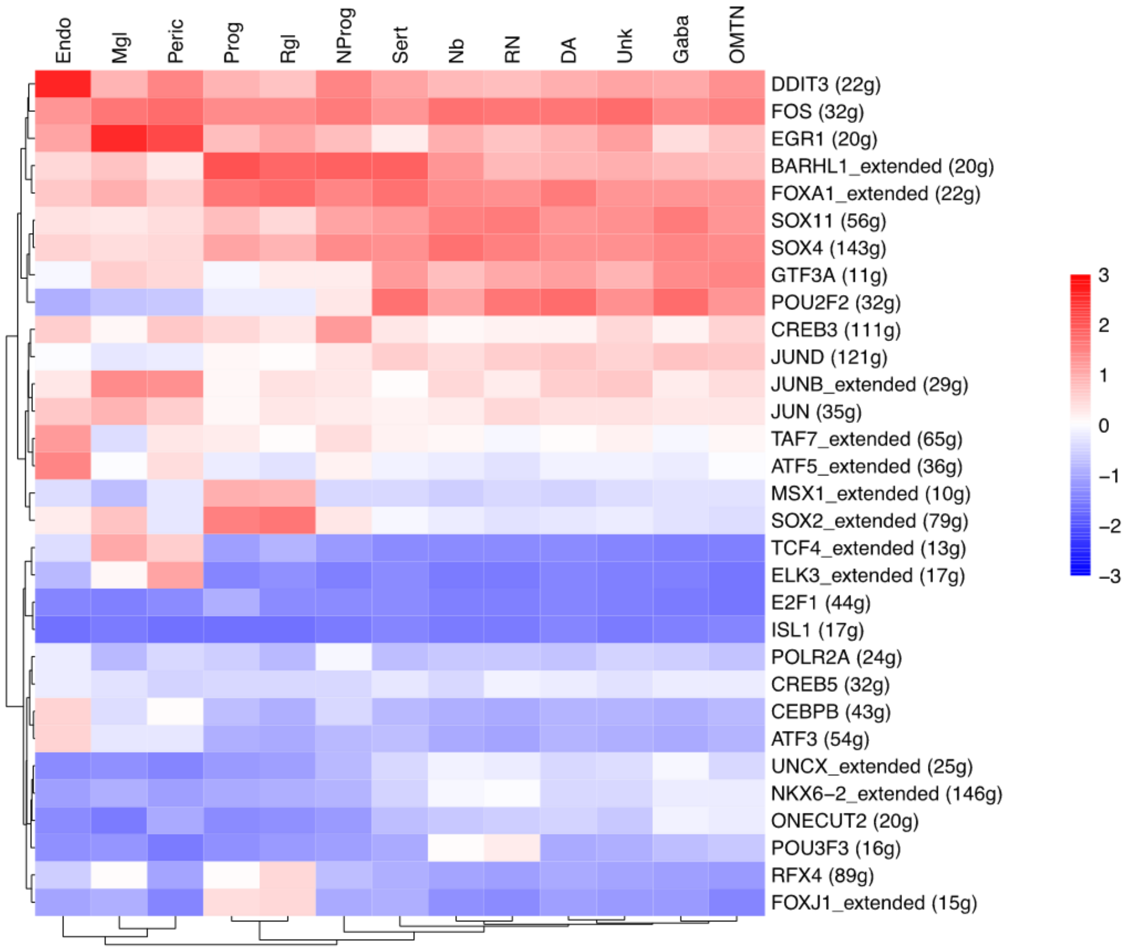
Endo
Mgl
Peric
Prog
Rgl
NProg
Sert
Nb
RN
DA
Unk
Gaba
OMTN
DDIT3 (22g)
FOS (32g)
EGR1 (20g)
BARHL1_extended (20g)
FOXA1_extended (22g)
SOX11 (56g)
SOX4 (143g)
GTF3A (11g)
POU2F2 (32g)
CREB3 (111g)
JUND (121g)
JUNB_extended (29g)
JUN (35g)
TAF7_extended (65g)
ATF5_extended (36g)
MSX1_extended (10g)
SOX2_extended (79g)
TCF4_extended (13g)
ELK3_extended (17g)
E2F1 (44g)
ISL1 (17g)
POLR2A (24g)
CREB5 (32g)
CEBPB (43g)
ATF3 (54g)
UNCX_extended (25g)
NKX6−2_extended (146g)
ONECUT2 (20g)
POU3F3 (16g)
RFX4 (89g)
FOXJ1_extended (15g)
3
2
1
0
−1
−2
−3

Figure S5 (cont’d)

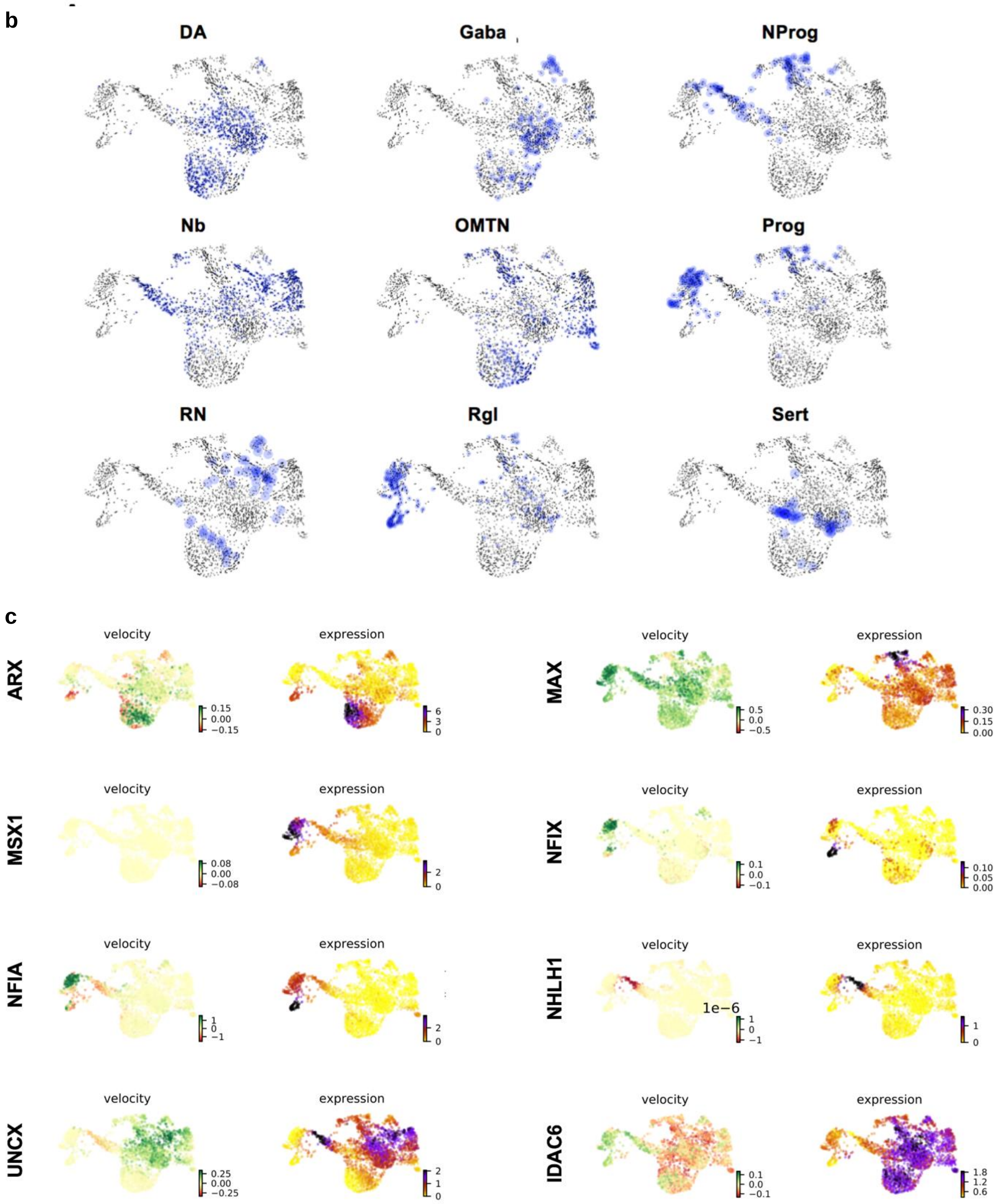

Figure S5. Critical regulators of cell identity and RNA velocity, related to Figure 5. (a) Critical regulators of cell identity constructed using SCENIC R (D79_WD). (b) Monochrome plots of cell types in D79_WD. (c) Phase portraits of key genes in D79_WD.

Table S1. Samples for single-cell RNA sequencing.

Sample ID	KH001	KH002	KH003	KH004	KH005
Days in culture	53	53	77	77	77 + 2
FTY treatment	untreated	acute (4Hr)	untreated	chronic (77 days)	chronic (77 days) withdrawal (2 days)
Sample name	D53_UT	D53_AC_FTY	D77_UT	D77_FTY	D79_WD
Cells sequenced by 10X chromium	5131	5,499	5,252	4,781	4,847
Mean reads per cell	79,997	74,256	72,722	73,999	77,290
Median genes per cell	3,963	3,735	4,342	3,603	4,684
Median UMI counts per cell	11,995	10,733	12,987	13,811	14,570
Total genes	25,960	25,772	25,316	24,780	25,090
Total reads	410,469,533	408,334,504	381,936,982	353,790,983	374,623,346

Table S2. Chemicals and materials.

Name	Vendor	Catalog Number
mTeSR Plus Basal Medium	Stemcell Technologies	1000276
ReLeSR	Stemcell Technologies	05873
StemPro Accutase Cell Dissociation Reagent	Gibco	A1110501
Y-27632 dihydrochloride (ROCK Inhibitor)	Tocris Bioscience	1254
Matrigel GFR Membrane Matrix	Corning	354230
KnockOut DMEM/F-12 Medium	Gibco	12660012
KnockOut Serum Replacement (KOSR)	Gibco	10828028
Glutamax Supplement	Gibco	35050061
Minimum essential media-nonessential amino acids solution (MEM-NEAA)	Gibco	11140050
2-mercaptoethanol	Gibco	21985023
Neurobasal Medium	Gibco	21103049
N-2 Supplement (100X)	Gibco	17502048
B-27 Supplement (50X), minus Vitamin A	Gibco	12587010
LDN193189	Stemgent	040074
SB431542	Tocris	1614
Recombinant Mouse Sonic Hedgehog / Shh (C25II) N-Terminus	R&D Systems	464-SH
Purmorphamine	Calbiochem	540220
CHIR99021	Stemgent	040004
BDNF Human	ProSpec	CYT-207
Ascorbic acid	Sigma	A-4034
GDNF	ProSpec	CYT-305
Dibutyryl-cAMP	Calbiochem	28745
TGF-b3	R&D Systems	243-B3
DAPT	Tocris	2634

Table S3. Antibodies used.

Antibody	Host species	Company	Cat. No.	Dilution
FOXA2	Rabbit	Invitrogen	720061	1:150
OTX2	Goat	Invitrogen	PA5-39887	1:100
TH	Rabbit	PelFreez	P40101-0	1:1000
GABA	Rabbit	Sigma	A2502	1:1000
MAP2	Chicken	Abcam	AB5392	1:2000
OPRM1	Rabbit	Sigma-Aldrich	AB5511	1:200
OPRK1	Mouse	Santa Cruz	SC-374479	1:50
OPRD1	Rabbit	Sigma-Aldrich	AB1560	1:100
Anti-Mouse-488	Donkey	Invitrogen	A-21202	1:500
Anti-Mouse-546	Goat	Invitrogen	A-11030	1:500
Anti-Mouse-647	Goat	Invitrogen	A32728	1:500
Anti-Rabbit-488	Goat	Invitrogen	A-11034	1:500
Anti-Rabbit-647	Donkey	Invitrogen	A-31573	1:500
Anti-Goat-568	Donkey	Invitrogen	A-11057	1:500
Anti-Goat-647	Donkey	Invitrogen	A-32849	1:500
Anti-Sheep-594	Donkey	Invitrogen	A-11016	1:500
Anti-Chicken-488	Goat	Invitrogen	A32931	1:500
Anti-Chicken-594	Goat	Invitrogen	A32759	1:500

Table S4. Primer sequences for real-time PCR.

Gene		Sequence (5'-3')
OCT4	Forward	GTGGAGGAAGCTGACAACAA
	Reverse	ATTCTCCAGGTTGCCTCTCA
NANOG	Forward	AAGGTCCCGGTCAAGAAACAG
	Reverse	CTTCTGCGTCACACCATTGC
TUJ1	Forward	GGCCAAGGGTCACTACACG
	Reverse	GCAGTCGCAGTTTTCACACTC
MAP2	Forward	TTGGTGCCGAGTGAGAAGAA
	Reverse	GGTCTGGCAGTGGTTGGTTAA
FOXA2	Forward	GGAGCAGCTACTATGCAGAGC
	Reverse	CGTGTTCATGCCGTTCATCC
LMX1A	Forward	ACGTCCGAGAACCATCTTGAC
	Reverse	CACCACCGTTTGTCTGAGC
TH	Forward	GGGCTGTGTAAGCAGAACG
	Reverse	AAGGCCCGAATCTCAGGCT
OXT2	Forward	CATGCAGAGGTCCTATCCCAT
	Reverse	AAGCTGGGGACTGATTGAGAT
DAT	Forward	TTTCTCCTGTCCGTCATTGGC
	Reverse	AGCCCACACCTTTCAGTATGG
PITX3	Forward	CCTACGAGGAGGTGTACCCC
	Reverse	CCCACGTTGACCGAGTTGA
PAX6	Forward	TCCACCCGGCAGAAGATTGTA
	Reverse	TGTCTCGGATTTCCCAAGCAA
TBR2	Forward	CACCGCCACCAAACTGAGAT
	Reverse	CGAACACATTGTAGTGGGCAG
GBX2	Forward	AAAGAGGGCTCGCTGCTC
	Reverse	ATC GCT CTC CAG CGA GAA
OPRM1	Forward	TACCGTGTGCTATGGACTGAT
	Reverse	ATGATGACGTAAATGTGAATG
OPRK1	Forward	CGTCTGCTACACCCTGATGATC
	Reverse	CTCTCGGGAGCCAGAAAGG
OPRD1	Forward	GCGGGAAAGCCAGTGACTC
	Reverse	TGCCCTGTTTAAGGACTCAGTTG

Table S5. Software and algorithms used.

Name	Reference	Source
Prism (v8.0)	GraphPad Software	N/A
JMP (v13.0)	SAS Institute	N/A
STAR (v2.5.2b)	Dobin et al., 2013[72]	https://github.com/alexdobin/STAR
Cell Ranger (v3.1.0)	10X Genomics	https://support.10xgenomics.com/single-cell-gene-expression/software/overview/welcome
R (v3.6.3)	The R Foundation	https://www.r-project.org
Python (v3.6)	Python Software Foundation	https://www.python.org/
Seurat (v3.1.1)	Satija et al., 2015[73]	http://satijalab.org/seurat/
Glmnet (v3.0.3)	Friedman et al., 2010[67]	https://glmnet.stanford.edu/
Monocle (v3.0)	Trapnell et al., 2014[74]<br>Qiu et al., 2017[75]	http://cole-trapnell-lab.github.io/monocle-release/
Velocyto (v0.17.16)	La Manno et al., 2018[70]	http://velocyto.org/
scVelo (v0.2.1)	Bergen et al., 2020[32]	https://scvelo.readthedocs.io/
SCENIC (v1.2.2)	Aibar et al., 2017[71]	https://aertslab.org/#scenic
ssGSEA	Wang et al., 2017[68]	https://doi.org/10.1016/j.ccell.2017.06.003
ComplexHeatmap (v2.5.6)	Gu et al., 2016[69]	https://jokergoo.github.io/ComplexHeatmap-reference/book/
Ingenuity Pathway Analysis	Qiagen Bioinformatics	https://www.qiagenbioinformatics.com/products/ingenuity-pathway-analysis/
